# Learning from mistakes—Assessing the performance and uncertainty in process‐based models

**DOI:** 10.1002/hyp.14515

**Published:** 2022-02-24

**Authors:** Moritz Feigl, Benjamin Roesky, Mathew Herrnegger, Karsten Schulz, Masaki Hayashi

**Affiliations:** ^1^ Department of Water, Atmosphere and Environment, Institute for Hydrology and Water Management University of Natural Resources and Life Sciences, Vienna Vienna Austria; ^2^ BGC Engineering Inc Toronto Canada; ^3^ Department of Geoscience University of Calgary Calgary Canada

**Keywords:** explainable, machine learning, process‐based modelling, stream temperature

## Abstract

Typical applications of process‐ or physically‐based models aim to gain a better process understanding or provide the basis for a decision‐making process. To adequately represent the physical system, models should include all essential processes. However, model errors can still occur. Other than large systematic observation errors, simplified, misrepresented, inadequately parametrised or missing processes are potential sources of errors. This study presents a set of methods and a proposed workflow for analysing errors of process‐based models as a basis for relating them to process representations. The evaluated approach consists of three steps: (1) training a machine‐learning (ML) error model using the input data of the process‐based model and other available variables, (2) estimation of local explanations (i.e., contributions of each variable to an individual prediction) for each predicted model error using SHapley Additive exPlanations (SHAP) in combination with principal component analysis, (3) clustering of SHAP values of all predicted errors to derive groups with similar error generation characteristics. By analysing these groups of different error‐variable association, hypotheses on error generation and corresponding processes can be formulated. That can ultimately lead to improvements in process understanding and prediction. The approach is applied to a process‐based stream water temperature model HFLUX in a case study for modelling an alpine stream in the Canadian Rocky Mountains. By using available meteorological and hydrological variables as inputs, the applied ML model is able to predict model residuals. Clustering of SHAP values results in three distinct error groups that are mainly related to shading and vegetation‐emitted long wave radiation. Model errors are rarely random and often contain valuable information. Assessing model error associations is ultimately a way of enhancing trust in implemented processes and of providing information on potential areas of improvement to the model.

## Graphical abstract

This study introduces the learning from mistakes workflow which was developed to analyse model errors of process‐based models. This analysis provides evidence for error‐process relationships which can be used to gain further insight and enhance trust in the applied model. The application of the workflow is shown in a case study. The application of the workflow is shown in a case study using a stream temperature model.

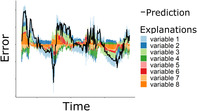

## 1 INTRODUCTION

There will always be model errors (residuals) apparent in any prediction, as models are only a crude representation of reality. While these errors might be random and the result of randomness in the investigated system (i.e., aleatoric uncertainty), there is also the possibility that they are related to the formulation of the model and thus to the chosen process representations (i.e., epistemic uncertainty or systematic uncertainty (Beven, 2016). This non‐randomness of residuals can occur due to (1) simplified, misrepresented or inadequately parameterised processes, (2) missing processes, (3) measurement errors. (1) Would also include any issues related to scaling (Blöschl & Sivapalan, 1995). Systematic measurement errors can be mitigated or excluded by adequate calibration procedures, frequent checks of the sensors and post‐processing. The other two potential error sources are more difficult to investigate and in a typical process‐based model application the occurrence of these errors is mainly identified using the investigators knowledge and experience with the model and the modelled system (Kirchner, 2006; Klemeš, 1986). While these errors might be negligible in some situations and with an experienced investigator, they potentially lead to unrecognised systematic errors and corresponding biassed conclusions. It is thus important to have an objective method to analyse model residuals to avoid systematic errors and enhance trust in the applied model. This is especially important if the results are used in some sort of decision‐making process (Jemberie, 2004), especially due to the dangers of uncritical application (Beven, 1989).

This study specifically focuses on model residuals and their relationship to processes represented in process‐based models. Therefore, we assume the model structure/formulation and all its parameters to be fixed. Hence, we aim to investigate how the performance of a fully set up process‐based model can be used to gain knowledge and to enhance trust in its usefulness for a given application.

While there exists a range of studies that applied model residual predictions to improve process‐based model outputs (e.g., Babovic et al., 2001; Demissie et al., 2009; Konapala et al., 2020; Wu et al., 2019; Xu et al., 2014; Young et al., 2017), to our knowledge only Jemberie (2004) investigated the relationship of model predicted residuals with observed variables to gain insight into process‐based models by using information theory and analysing machine learning approaches to predict model errors. He developed a two‐step approach that included (1) an assessment of the information content of model residuals and a selection of related observed variables using the average mutual information measure and (2) the prediction of model errors using selected observed variables and a neural network. When combining (1) and (2) with physical insight, patterns of errors can potentially be traced back to physical processes. Based on the same ideas as formulated in Jemberie (2004), we here present an approach that allows to assess not only the general relationship between errors and observed variables, but also the relationship between each particular model error to observed variables to further gain physical insight from model errors. This is made possible by the recent developments in explainable machine‐learning methods (Molnar et al., 2020), which result in several methods to estimate variable importance for machine‐learning models.

This study presents the learning from mistakes (LFM) workflow for analysing errors of process‐based models as a basis for relating them to process representations. The evaluated approach consists of: (1) training a machine‐learning error model using the input data of the process‐based model and other available variables, (2) estimation of local explanations (i.e., contributions of each variable to an individual prediction) for each predicted model error using SHapley Additive exPlanations (SHAP, Lundberg et al., 2017, 2020) in combination with principal components (PC), and (3) clustering of SHAP values of all predicted errors to derive groups with similar error generation characteristics. By analysing these groups of different error/variable association, hypotheses on error generation and corresponding processes can be formulated.

To show the general applicability of our proposed methods for assessing error‐variable relationships, we apply the LFM workflow in a case study of an alpine stream in the Canadian Rocky Mountains with the process‐based stream water temperature model HFLUX (Glose et al., 2017). Water temperature in first‐order streams are sensitive to a multitude of energy exchange processes between water and air and water and sediment, including radiation, turbulent heat fluxes, bed conduction, and groundwater exchange (Moore et al., 2005). Therefore, this case study serves as a useful example to demonstrate the model sensitivity to errors in various model variables representing different physical processes. It is used to propose hypotheses for error generation and define situations of low and large uncertainty of model prediction. The model and study site were chosen due to our detailed knowledge of the study area, the measurements and the model implementation from a previous study (Roesky & Hayashi, 2022).

In the following, we introduce the LFM workflow for objectively assessing the relationship between model errors and available variables. The originality of the study includes (1) a novel approach that allows model developers, users and stakeholders to gain further insight into the applied process‐based model, (2) an extension to SHAP values to estimate local explanations for correlated variables, and (3) an application in a case study for simulating water temperature.

## 2 MATERIALS AND METHODS

The following section is separated into three distinct parts describing (1) the LFM workflow, (2) PCA SHAP and (3) the case study with the process‐based stream water temperature model HFLUX. The first part includes a depiction of the workflow which is generally applicable for any process‐based model, the second part contains a description of our proposed extension to SHAP values for correlated datasets and the third part includes modelling choices that are specific to the presented case study.

### 2.1 LFM workflow

#### 2.1.1 Preliminary steps

The LFM workflow assumes a fixed model setup, model residuals and a set of variables that are potentially associated with the model residuals. These variables will in most cases consist of the model input variables as well as any available variables that could be relevant for the modelled processes. A prerequisite for deriving insights from model residuals is their association with these variables, or more generally—their non‐randomness. Therefore, before starting the LFM workflow, a linear regression model should be applied together with a *F*‐test to check for significance association between model residuals and variables. In case there are no significant associations, hence no linear relationships, a very simple ML‐model (e.g., random forests) can be used to check if at least some of the model error can be predicted. If this still does not produce a significantly improved prediction, the assumption of non‐randomness might not hold.

**FIGURE 1:**
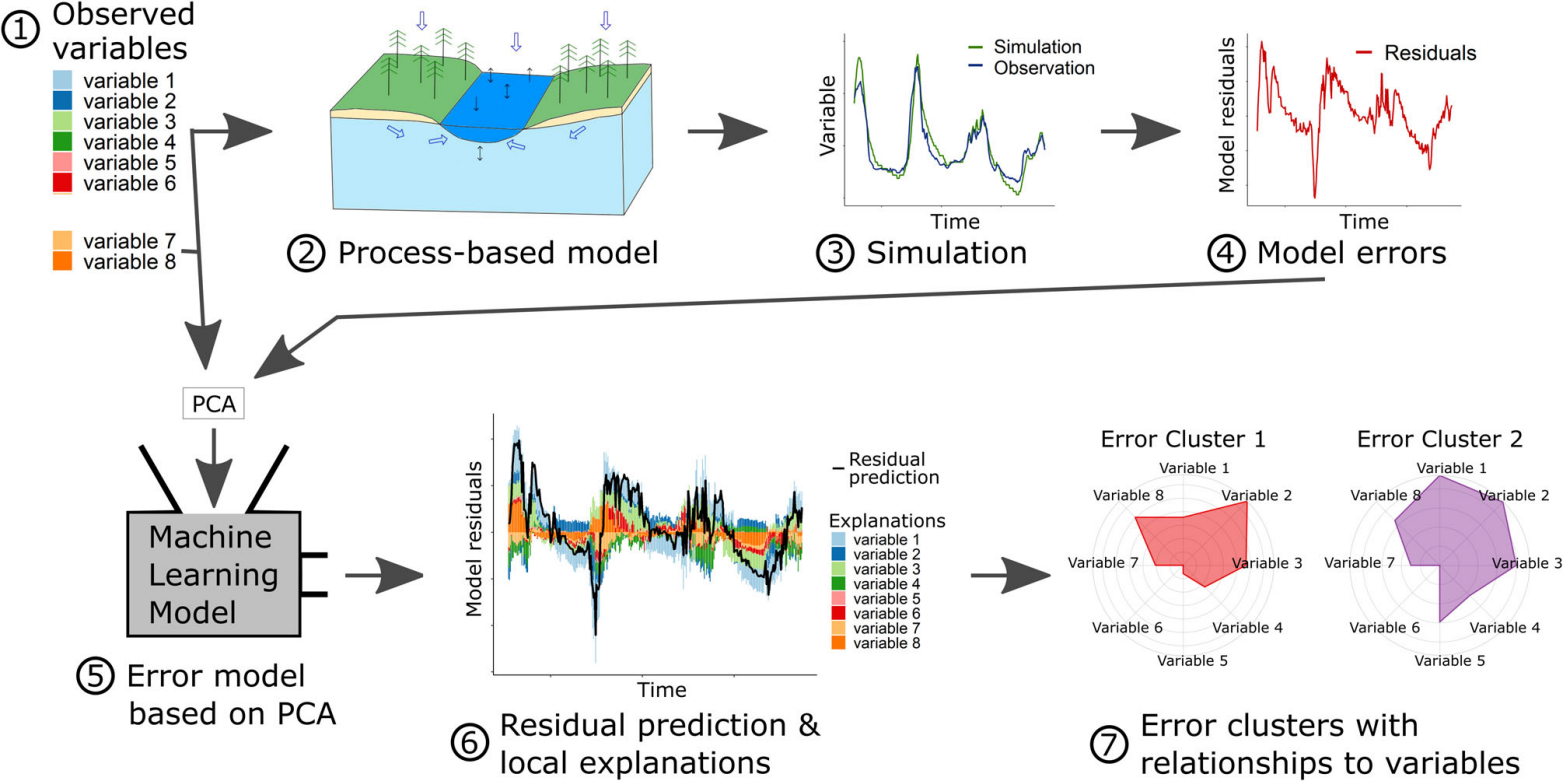
Learning from mistakes workflow. ① The available observations. ② The process‐based model that should be investigated. ③ The simulation results compared to the observed values. ④ The resulting model residuals. ⑤ The ML‐model that is used to predict model residuals from PCA transformed observed variables. ⑥ The predicted residuals and the local explanations derived from the ML‐model. ⑦ Importance of single variables for a cluster derived from the local explanations. The value of each variable in these plots shows the median local explanation for the corresponding cluster

The LFM workflow is shown in Figure 1 and starts with the process‐based model application and model residual generation (Figure 1 part 1–4). In this study we define residuals as values resulting from subtracting observed values from predicted values, hence positive residuals imply overestimation and negative residuals imply underestimation.

#### 2.1.2 Machine learning models for residual prediction

After deriving residuals, we can set up a machine‐learning model (Figure 1 part 5) that predicts model residuals from available observed variables. However, before using the observations as input for the ML‐model, a principal component analysis (PCA, Pearson, 1901) is applied to derive their principal components (PCs).

PCA converts possibly correlated variables in a set of linearly uncorrelated variables. Deriving uncorrelated features is necessary, as dependency structures between input variables can potentially lead to biassed variable usage for predictions in the applied ML‐model, that is, in the extreme case variables are not used by the model since other highly correlated variables are used instead. The PCA is applied on standardised inputs, that is, subtracting the mean of the values of a variable and divide the resulting centred values by their standard deviation. The resulting PCs are linear combinations of these standardised inputs. Hence, the *i*th PC can be written as,

(1) $${\mathrm{PC}}_{i}=\sum_{j=1}^{K}\gamma_{\mathrm{ij}}x_{j},$$

where $x_{j}$ denote the standardised input variables and $\gamma_{\mathrm{ij}}$ their factor loadings. Factor loadings of PCs of standardised inputs are the correlation coefficients between variables and PCs. A PCA results in an equal number of PCs than original variables, which can be directly used as input features for the ML‐model.

Originally, some PCA properties assume normality of the input variables, but a violation of normality should not influence the usefulness of PCA (Jolliffe, 2002). Since, the reason for applying PCA in the LFM workflow is to create independent variables, this can also be checked in case non‐linear dependency structures in the data are believed to exist. Possible measure of dependency that could be applied are Hoeffding's D (Hoeffding, 1948) and the maximum information criteria (Reshef et al., 2011).

Setting up an adequate ML‐model for residual prediction is arguably the most challenging part of this workflow but will in most cases be solvable with ready‐to‐use models from widely used ML‐libraries. The supporting information (Data S1) include a brief guide on how to choose a ML‐model for residual prediction.

#### 2.1.3 Computing local explanations

**FIGURE 2:**
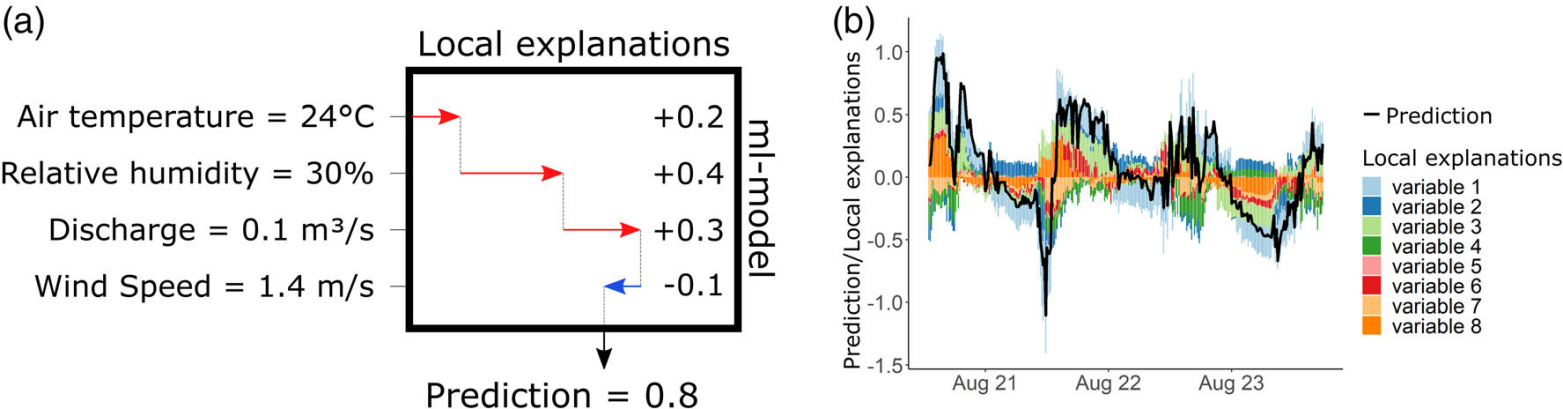
(a) Local explanations for 4 variables of a single prediction (inspired by Lundberg et al., 2020). (b) Local explanations of 8 variables for a time series of predictions. Each individual time step of this time series could also be depicted as in (a). In case lagged versions of a variable are used, their local explanations can be summed up to represent the overall impact of the lagged variable

A ML‐model that can adequately predict model residuals shows that there is valuable information contained in the available data. To further gain insight in the dependencies between individual residuals and ML‐model inputs, it is necessary to derive local explanations, that is, contribution of each ML‐model input for an individual prediction (Baehrens et al., 2010; Lundberg et al., 2020). Local explanations reveal the impact of each input variable of an ML‐model on the resulting prediction. Figure 2a shows a simple example of the local explanations of four variables and the corresponding prediction value. In case lagged versions of variables are used as well, for example, air temperature of the previous days, these will have their own local explanation values that can be aggregated to depict the overall impact of the lagged variable. Figure 2b shows an example of local explanations for eight variables for a time series of predictions. The next step of the LFM workflow consists of computing local explanations for predicted residuals as shown in Figure 1 (part 6).

Recent development in interpretable machine‐learning resulted in multiple methods for local explanations (see e.g., Molnar, 2019; Molnar et al., 2020). While multiple methods exist, the LFM workflow was developed using SHapley Additive exPlanations (SHAP, Lundberg et al., 2017, 2020) as they are applicable to any type of ML‐model, have a solid theoretical foundation in game theory (Molnar, 2019) and their interpretation is consistent with human intuition (Lundberg et al., 2017). The SHAP method and its extension for correlated input variables developed for the LFM workflow—PCA SHAP—will be described in detail in Section 2.2.

#### 2.1.4 Error clusters

This final step aggregates the local explanation values into comprehensible error groups, which are the basis for deriving hypotheses on error cluster to processes relationships. It consists of finding clusters with similar underlying error generation associations, that is, clusters of residuals with similar local explanation patterns (see Figure 1; part 7). This can be achieved by a range of clustering algorithm, for example, Hierarchical clustering (Ward, 1963) or k‐means (MacQueen, 1967), in combination with methods to assess cluster separation, where several possibilities exists (e.g., Davies & Bouldin, 1979; Dunn, 2008; Rousseeuw, 1987). To find the optimal number of clusters and the most adequate clustering method, we propose to assess the performance of multiple cluster algorithms with different numbers of clusters using silhouette coefficients (Rousseeuw, 1987). Silhouette coefficients are a measure that describes how similar an object is to its cluster. They can range from −1 to 1 and are defined as the normalised difference between the mean distance of a point to its own cluster and the mean distance of a point to its neighbouring cluster. High positive values indicate adequate separation, negative values indicate poor cluster quality.

To this point, all steps of the LFM workflow can generally be applied to any model setup. However, understanding the relationships between modelled processes and errors and deriving error generation hypotheses is strongly depending on the model and the type of application. A model developer will most likely be interested in different process‐error relationships compared to a user who wants to show that only a certain type of process is related to model errors and thus the model can be used for her/his specific use case. In the following case study, we provide examples on how to approach this task, but we also emphasise that even though the LFM workflow results in evidence for error‐process relationships, a certain understanding of the model and its processes is necessary to interpret these.

### 2.2 PCA SHAP

SHAP is based on shapley values (Shapley, 1953), a concept in game theory that estimate the marginal contributions of every player on the outcome of a game. In the case of predictive modelling, the game is predicting the model outcome and the players are the ML‐model inputs. SHAP is an additive feature attribution method and estimates a linear explanation model g of the form,

(2) $$g\left(x\right)=\phi_{0}+\sum_{i=1}^{K}\phi_{i}\left(f,x\right),$$

where $x\in {\mathbb{R}}^{K}$ denotes the inputs of the ML‐model, f denotes the ML‐model, $\phi_{0}\in \mathbb{R}$ is the base value, $\phi_{i}\in \mathbb{R}$ the SHAP values for each input variable computed using *f* and *x*, and *K* the total number of input variables. The base value reflects the ML‐model value we would predict without knowing any input variables.

SHAP values are estimated in a way that results they satisfy the local accuracy property $f\left(x\right)=g\left(x\right),$ meaning that the sum of base values and SHAP values is always equal to the predicted value. A detailed definition and explanation of how SHAP values are computed is given in Lundberg et al. (2017).

SHAP in its original form is not able to handle correlated features and can potentially result in highly inaccurate explanations (Aas et al., 2021). Since environmental variables are often highly correlated, we developed a method to estimated explanations from dependent features based on principal components. Recently Aas et al. (2021) were the first to develop a SHAP extension to solve the problem of analysis of correlated variables, which is based on implementing the estimation of an appropriate marginal distribution of dependent variables. While this shows promising results in their applications, it adds other assumptions to the SHAP value estimations and restricts to using the kernel SHAP implementation instead of the faster Tree SHAP (Lundberg et al., 2020) or Deep SHAP (Lundberg et al., 2017) implementation. Hence, we propose PCA SHAP values as an extension to the original SHAP values, which allow to use already available faster SHAP methods, while correcting them for feature dependencies.

After computing SHAP values for the PCs that were used as input for the ML‐model, SHAP values reflecting the contribution for each original observed variable are computed by multiplying them with their corresponding PC loadings. Thus, the PCA SHAP value of one variable ${\tilde{\phi }}_{j}$ for a single prediction can be estimated by,

(3) $${\tilde{\phi }}_{j}=\sum_{i=1}^{K}\gamma_{\mathrm{ij}}\phi_{i}\left(f,x\right),$$

where $\phi_{i}$ denotes the SHAP value of the *i*th PC and $\gamma_{\mathrm{ij}}$ the factor loading of the *i*th PC and *j*th variable. These PCA derived SHAP values reflect the importance of each variable without being influenced by feature correlation. This is especially important in environmental applications, where in most cases observations are highly correlated. However, deriving these new estimates for local explanations leads to the loss of the local accuracy property, that is, the sum of PCA SHAP values will not necessarily be equal to the prediction. This loss of local accuracy is due to the fact the PCA SHAP values of different variables can potentially contain overlapping information. The PCA SHAP value of each variable still reflect its influence on the prediction and is comparable to PCA SHAP value of the other variables. Thus, PCA SHAP values, as the original SHAP values, still reflect the fraction of the prediction value that is attributed to a variable.

### 2.3 Case study: Stream water temperature modelling

This case study assesses the uncertainties and error‐process relationships of the process‐based stream water temperature model HFLUX in an application for simulating water temperature in a small headwater stream in the Canadian Rocky Mountains. The corresponding field work, data collection, data preparation and model setup are described by Roesky and Hayashi (2022). The following will give a brief overview of the study area, the model setup and all specifics of the LFM application.

#### 2.3.1 Study site and data

**FIGURE 3:**
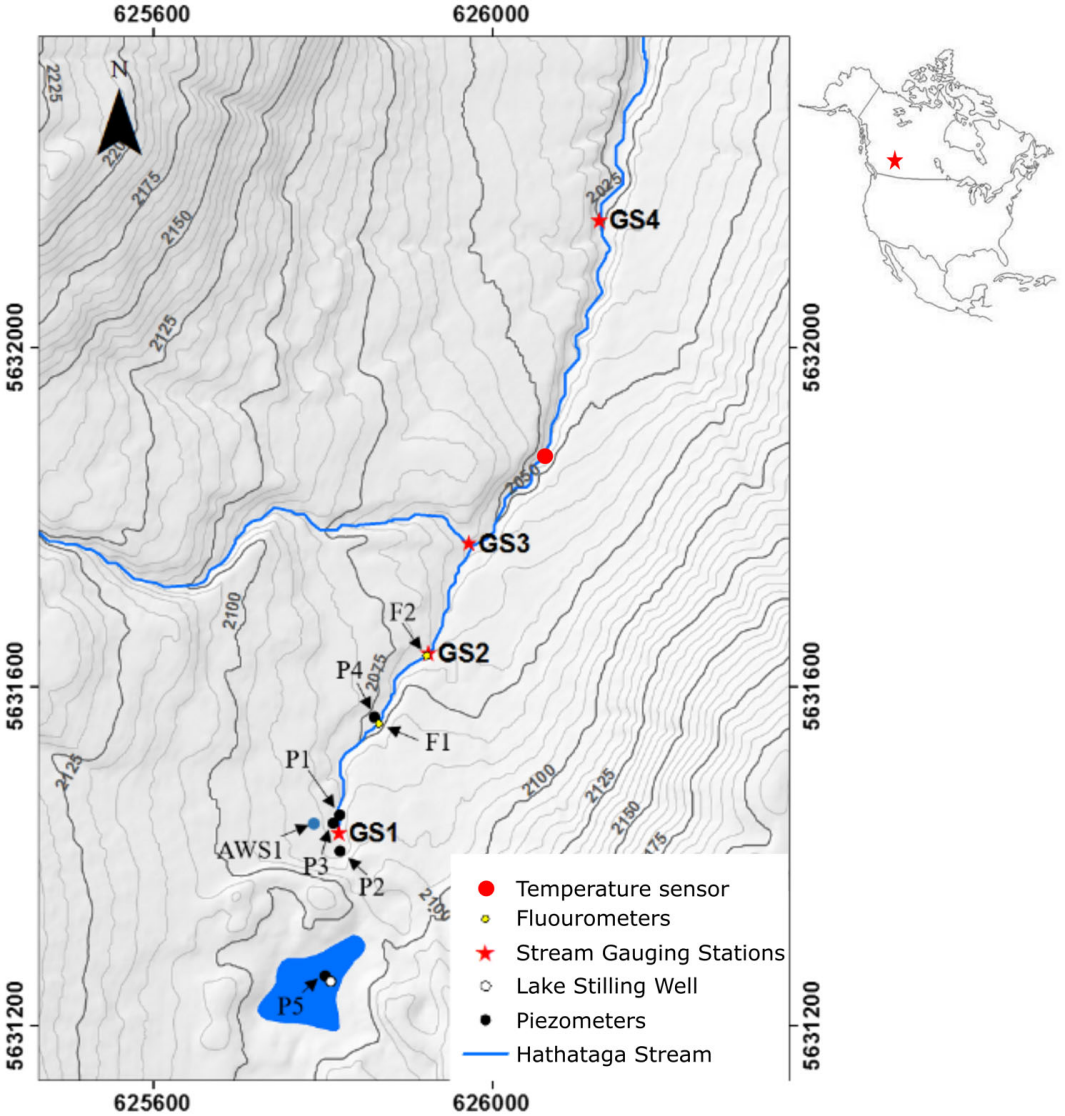
Topography of the study area with locations of the temperature sensor that is evaluated in this study, stream gauging stations (GS1–GS4), piezometers (P1–P5), fluorometers (F1, F2) and the Hathataga Lake stilling well. Dark contour lines are drawn at 25 m intervals and light contours at 5 m intervals. Datum: NAD83, zone 11 N. the map in the upper right corner shows North America with the catchment location depicted as a red star. Modified after Roesky and Hayashi (2022)

The study site is a headwater stream located on the eastern slopes of the Canadian Rocky Mountains in the Fortress Ski Area (50°49′14″, −115°12′46″). It is a small alpine stream with a catchment area of 3.45 km^2^, an elevation range of 2034–2900 m above sea level., a mean air temperature of −1.5°C (in 2019) and a maximum discharge of 0.55 m^3^s^−1^ (in 2019). An overview of the stream, gauging stations (GS), meteorological station (AWS) and topography is shown in Figure 3. The stream starting point at gauging station 1 (GS1, Figure 3) is a merging point of three perennial springs (next to the piezometers P1‐3, Figure 3). HFLUX is set up to simulate water temperature from GS1 to GS4, a reach of 850 m.

Data are available from 12:00, 8 August to 12:00, 26 August 2019 (Mountain Daylight Time, UTC −6). Stream water temperature was measured in 15 min intervals at 11 points in the stream. Figure 3 shows the measurement point that was finally used for the error model in this study for the position of the other points refer to (Roesky & Hayashi, 2022). Discharge was measured continuously at the four gauging stations (GS_*i*_, Figure 3). Meteorological data was available from an automatic weather station (AWS1, 2084 m.a.s.l., Figure 3) installed by the University of Saskatchewan located in the meadow next to the three headwater springs. It measured air temperature, relative humidity, shortwave and longwave radiation, canopy temperature and wind speed. The western tributary (at GS3, Figure 3) was treated as distinct water source with measured discharge and water temperature.

#### 2.3.2 Stream water temperature modelling

To model stream water temperature along the study stream, the one‐dimensional heat transfer model HFLUX (Glose et al., 2017) was used. HFLUX solves the differential equation:

(4) $$\frac{\partial \left(AT_{w}\right)}{\partial t}+\frac{\partial \left(QT_{w}\right)}{\partial x}=Q_{L}T_{L}+\frac{Wq_{t}}{\rho_{w}c_{w}},$$

where *A* (m^2^) is the cross‐sectional area of the channel, *Q* (m^3^s^−1^) the discharge, $T_{w}$ (°C) the stream water temperature, *Q*_L_ (m^3^s^−1^m^−1^) and *T*_L_ (°C) the lateral inflow and the inflow temperature, *W* (m) the width of the stream, *q*_t_ (W m^−2^) the total energy flux to the stream, *ρ*_w_ (kg m^−3^) the density of water and *c*_w_ (J kg^−1^°C^−1^) the specific heat of water. The total energy flux is the sum of all incoming energy fluxes, which are listed in Table 1 together with all measured and estimated variables that are used to derive them in HFLUX.

**TABLE 1:** Energy fluxes included in HFLUX and their relation to measured and estimated variables

Energy flux (W m^−2^)	Variables (measured)	Variables (estimated or assumed)
Net shortwave radiation	Measured incoming shortwave radiation	Shading, Albedo
Net longwave radiation	Measured incoming longwave radiation, air temperature, water temperature	View to sky coefficient
Latent heat flux	Relative humidity, wind speed, air temperature, water temperature	Empirical constants for height of measurement
Sensible heat flux	Air temperature, relative humidity, sea level, water temperature	
Precipitation flux	Precipitation	
Bed conduction	Bed temperature, water temperature	Sediment thermal properties
Advection	Tributary inflow temperature, discharge	Groundwater temperature

*Note*: The empirical constants for height of measurement refers to constants that depend on the heights at which wind speed and vapour pressure are measured (Dingman, 1994; Glose et al., 2017).

The chosen modelling time step and distance step were 1 m and 1 min, respectively. HFLUX was calibrated for 12:00 August 14–12:00 August 20. Variables/parameters estimated during the calibration process included spatially variable, temporally constant shading factor, constant groundwater temperature and the view to sky coefficient. All of them are assumed to be constant between each sub‐reach (i.e., between temperature sensors), which are on average 90 m long with a mean travel time of approximately 4 min. HFLUX approximates canopy temperature using air temperature.

#### 2.3.3 LFM setup

Initial analysis showed that model residuals of the 11 different stream temperature measurement points are highly correlated (correlation median = 0.82), thus we selected the measurements of the temperature sensor located at 640 m downstream of GS1 (red point in Figure 3) to compute model residuals. This point is located far enough from the starting point with known boundary conditions that the water temperature is notable different from the incoming stream water temperature and not yet too far from the meteorological station providing input data for HFLUX so that its observations are still reflecting its micro‐climatic conditions. Since stream temperature measurements were only available in 15 min intervals, the residuals were computed with the predicted stream temperature of the corresponding minute.

As input for the ML error model, we used all available time series data in the same 15 min interval, including discharge and the data of the meteorological station. For these variables we also computed lagged versions. The number of lags were estimated during ML‐model optimisation. Instead of using the discrete cyclical variable “hours of the day” as ML‐model input, we computed a sine/cosine transformed version with $\text{hour}\left[\sin\right]=\sin\left(2\times \pi \times \frac{\text{hour}}{24}\right)$ and $\text{hour}\left[\cos\right]=\cos\left(2\times \pi \times \frac{\text{hour}}{24}\right).$ This would allow to predict time dependent influences on model residuals that are not represented by the other input variables. All variables were transformed into an equal number of PCs by performing a PCA, before applying them in the ML‐model.

The error model was chosen to be XGBoost (Chen & Guestrin, 2016), a regression tree based boosting algorithm. This choice was made based on our experience with stream temperature prediction from a previous study (Feigl, Lebiedzinski, et al., 2021), which showed that the performance of most of the tested ML‐models mainly dependent on the input data and the hyperparameter optimisation. Only recurrent neural networks (RNNs) showed different behaviour as its structure already allows for sequence of inputs instead of tabular data. Preliminary results however showed that RNNs (LSTMs and GRUs) did have a lower performance compared to XGBoost. We trained it on 13 days of data with a 5 times repeated 10‐fold cross validation and tested it on different randomly selected time steps distributed over the whole study time period with a total length of 5 days. The model hyperparameters were estimated by Bayesian hyperparamter optimisation (Kushner, 1964; Močkus, 1989; Snoek et al., 2012) with 50 iterations. The optimised hyperparameters and their bounds are listed in Appendix A. This was repeated for different lags of input variables in the range of 0–12 time steps (each 15 min). No lag combinations were included, that is, each lag of a variable is represented by an additional column in the input data. The chosen loss function was the root mean squared error (RMSE).

SHAP values were computed using the shap python library (github.com/slundberg/shap). After computing the PCA derived SHAP values for each original input variable, all lagged SHAP values and the sine/cosine hour variables were summed up to represent the overall importance of the particular variable. Due to overlapping information contained in the lagged variables, summing can potentially overestimate their importance in comparison to non‐lagged variables (i.e., hour variables). Therefore, we scale the summed PCA SHAP values of the hour variables by multiplying them with 2/*n*lags (=4.5). This representation is used for plotting and summary statistics, while the original derived PCA SHAP values (before summing lags and sine/cosine variables) are used for clustering. Three clustering algorithms, (1) k‐means (MacQueen, 1967) and two types of hierarchical clustering (Ward, 1963), namely (2) complete linkage hierarchical clustering and (3) ward hierarchical clustering were selected. They were applied to cluster PCA derived SHAP values into error groups, with a possible number of clusters in the range 2–15. The best clustering result was chosen by assessing the mean silhouette coefficient.

The properties of all error clusters are investigated and used to propose hypotheses on error generation. This is followed by a test in which the model is adapted without further calibration to minimise these process related errors.

## 3 RESULTS

### 3.1 Error model

**FIGURE 4:**
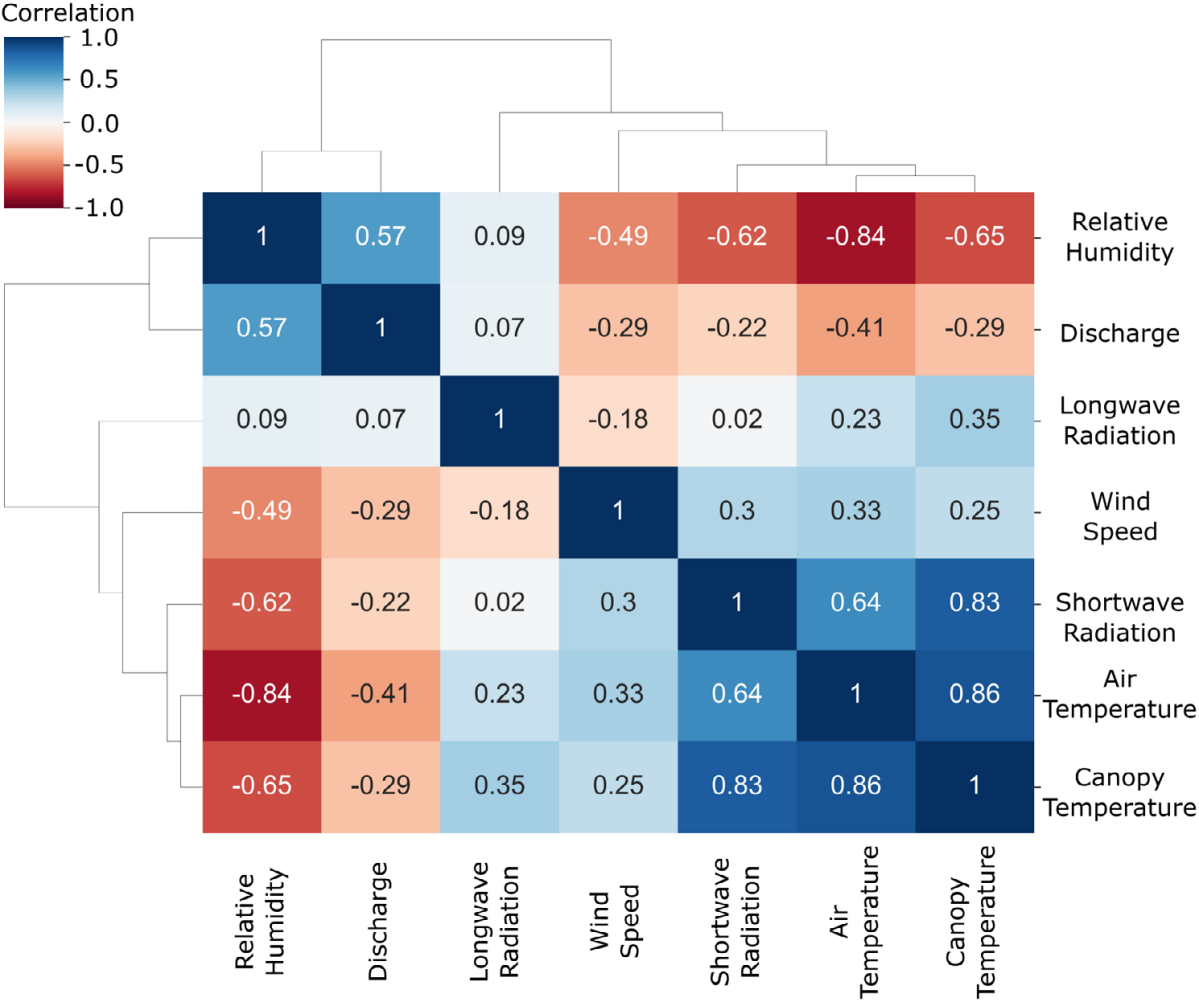
Correlation matrix of available input variables, with grouping by complete linkage hierarchical clustering

Figure 4 shows the Pearson correlation coefficients of all variables used as input in the error model. The dendrogram outside of the correlation matrix shows the grouping of a complete linkage hierarchical clustering. Except for longwave radiation with a maximum correlation of 0.35, correlation values show that available variables are not independent. Especially shortwave radiation, air temperature, canopy temperature and relative humidity are highly correlated with a mean absolute correlation of 0.74. Also discharge and relative humidity have a correlation of 0.57, reflecting the daily cycle in discharge present in the stream. While this was to be expected, it shows the importance of considering variable dependencies when estimating local explanations. To further illustrate the interactions of these variables, we included time series of all variables for the first week of the study period in the Data S1 (supporting information S3).

**FIGURE 5:**
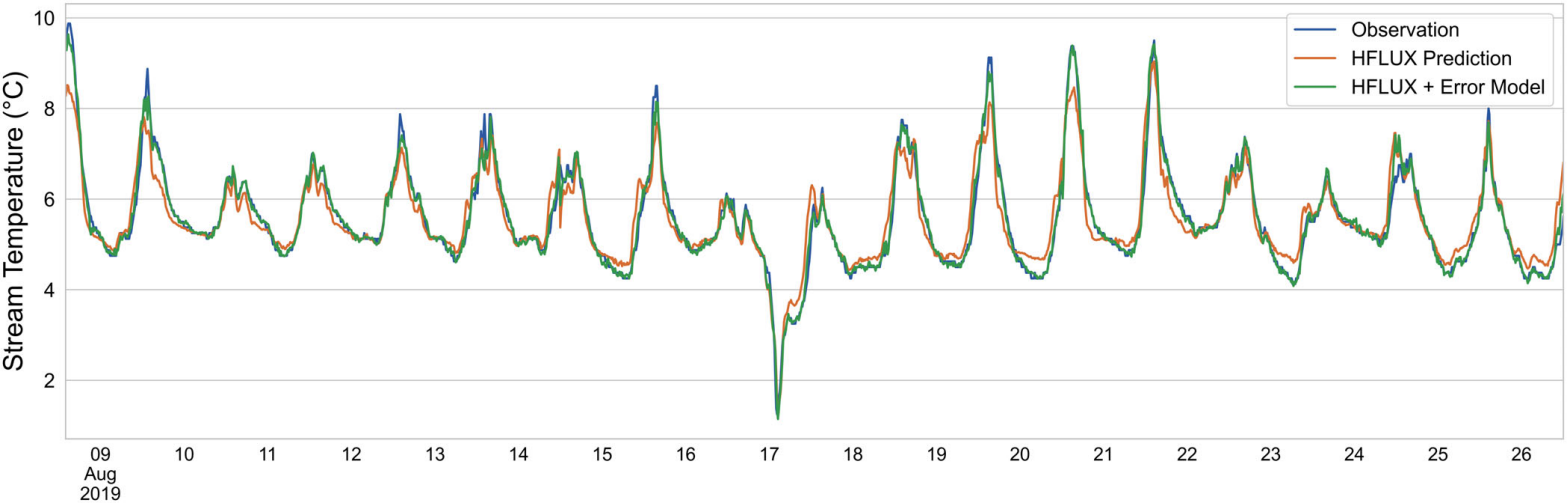
Comparison of observed and predicted time series of stream temperatures. Observed values are shown in blue, HFLUX predicted values in orange and HFLUX predictions with the additional error model are shown in green

The results of the original HFLUX prediction and the HFLUX with the ML‐model prediction is shown in Figure 5. The optimised ML‐model is using eight lags, that is, data of the two previous hours in addition to the current time step, for predicting HFLUX errors. While the original HFLUX prediction had a RMSE of 0.378°C, the HFLUX error model combination has an overall RMSE of 0.145°C. Cross validation, training and test RMSE of the error model were 0.232, 0.118 and 0.222 respectively. Looking at Figure 5, we see that the combined model prediction fits well with observations. Nevertheless, some stream temperature peaks are still underestimated and the stream temperature overestimation on the double peak on August 24 can also not be predicted by the ML‐model with the available data.

### 3.2 Error‐variable relationships and clustering

**FIGURE 6:**
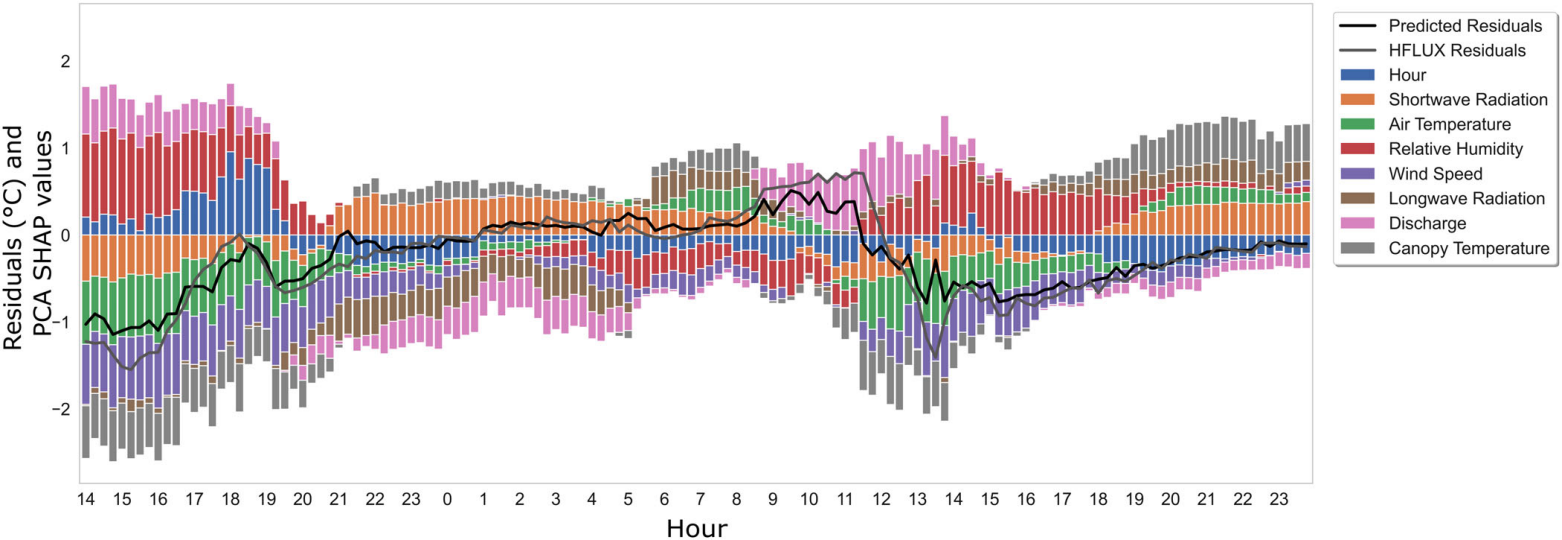
Time series of ML‐model predicted residuals, HFLUX model residuals (°C) and PCA SHAP values for the 8/9 August 2019. The PCA SHAP values of hour consists of the sum of ${\text{hour}}_{\sin}$ and ${\text{hour}}_{\cos},$ and the PCA SHAP values of all other variables consist of the sums of PCA SHAP values of the variables and their lagged versions. Furthermore, the hour variable is scaled by a factor of 2/9 to avoid an underestimation of its importance in this aggregated representation

An example of the PCA SHAP values plotted together with the HFLUX model residuals and the predicted residuals for August 8–9 is shown in Figure 6. For these days differences in the error patterns during night and daytime can be observed. For understanding these patterns, it is also necessary to keep in mind that even an input variable value of 0 can have a PCA SHAP value that is not zero, as they are still used for prediction, for example, shortwave radiation at night. It is also notable that the variable hour has usually low importance, pointing to the fact that there are only few systematic errors that cannot be described with available other variables. While this representation gives insights into the error‐variable relationships, it is hard to interpret due to its complexity, especially when examining the full study time period. This motivates the use of cluster analysis to aggregate these results to find distinct groups of errors that can be further examined.

**FIGURE 7:**
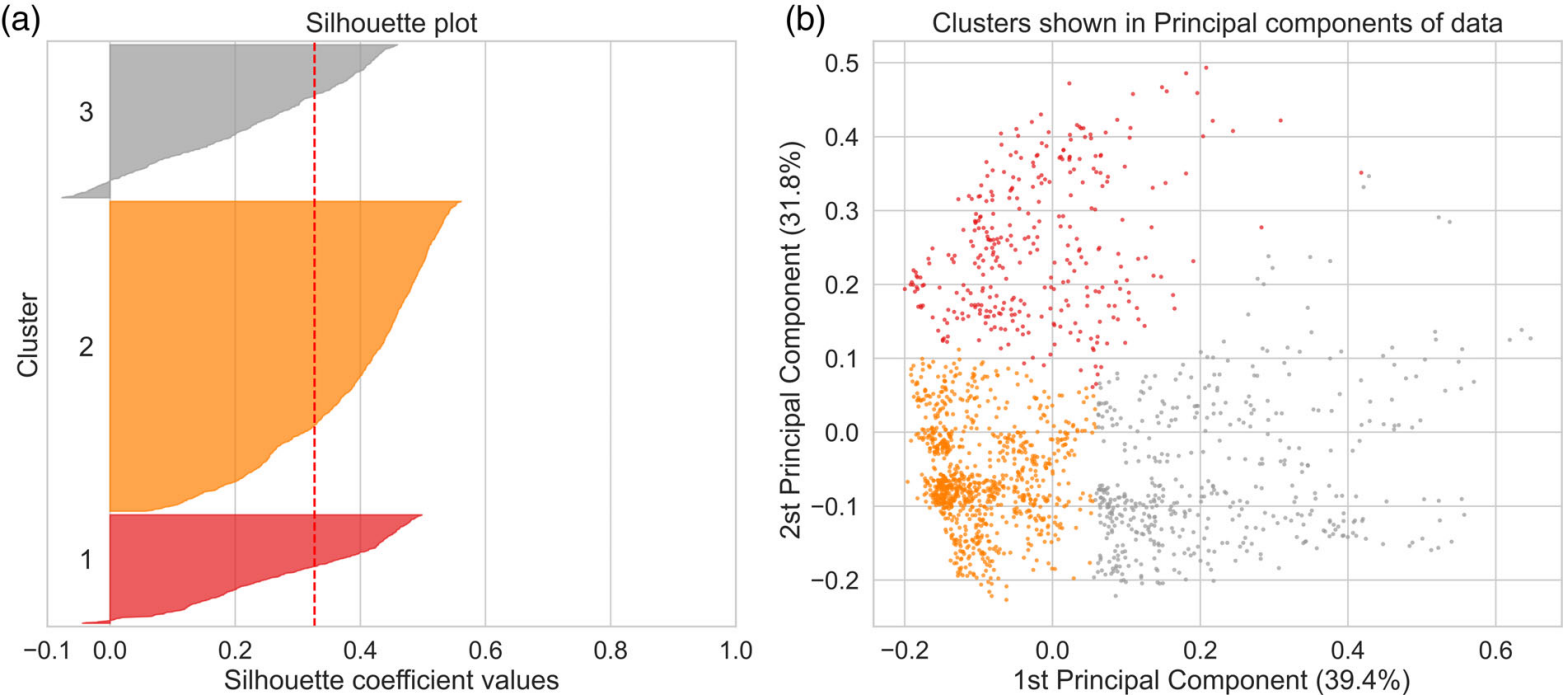
Cluster analysis results for the algorithm and number of clusters with the highest mean silhouette coefficient. (a) The silhouette coefficient values for all points of the three clusters, with the overall mean shown as red dashed line. (b) scatter plot with all data points shown with their position defined as their values of the first and second PC. Each point represents the PCA SHAP values of one time step of the study time period. The percentage in the axis description shows the amount of total variance that can be explained by each PC

Clustering results showed the best performance in terms of silhouette coefficient values for the k‐means algorithm with 3 clusters, with a mean silhouette coefficient value of 0.35. Figure 7a shows all silhouette coefficient values for all points of the 3 clusters, with the mean value shown as red dashed line. Figure 7b shows the cluster positions on the first two PCs of the data points. Each point represents the PCA SHAP values of one time step. These results illustrate the cluster quality and the position of the clusters in the high‐dimensional space of PCA SHAP values.

### 3.3 Error clusters and hypotheses

In this section we examine each of the derived error clusters and propose hypotheses of their relation to processes. The PCA SHAP values for the full study period can be found in the Data S1 (supporting information S2).

#### 3.3.1 Cluster 1: Decreasing discharge and morning air temperatures

**FIGURE 8:**
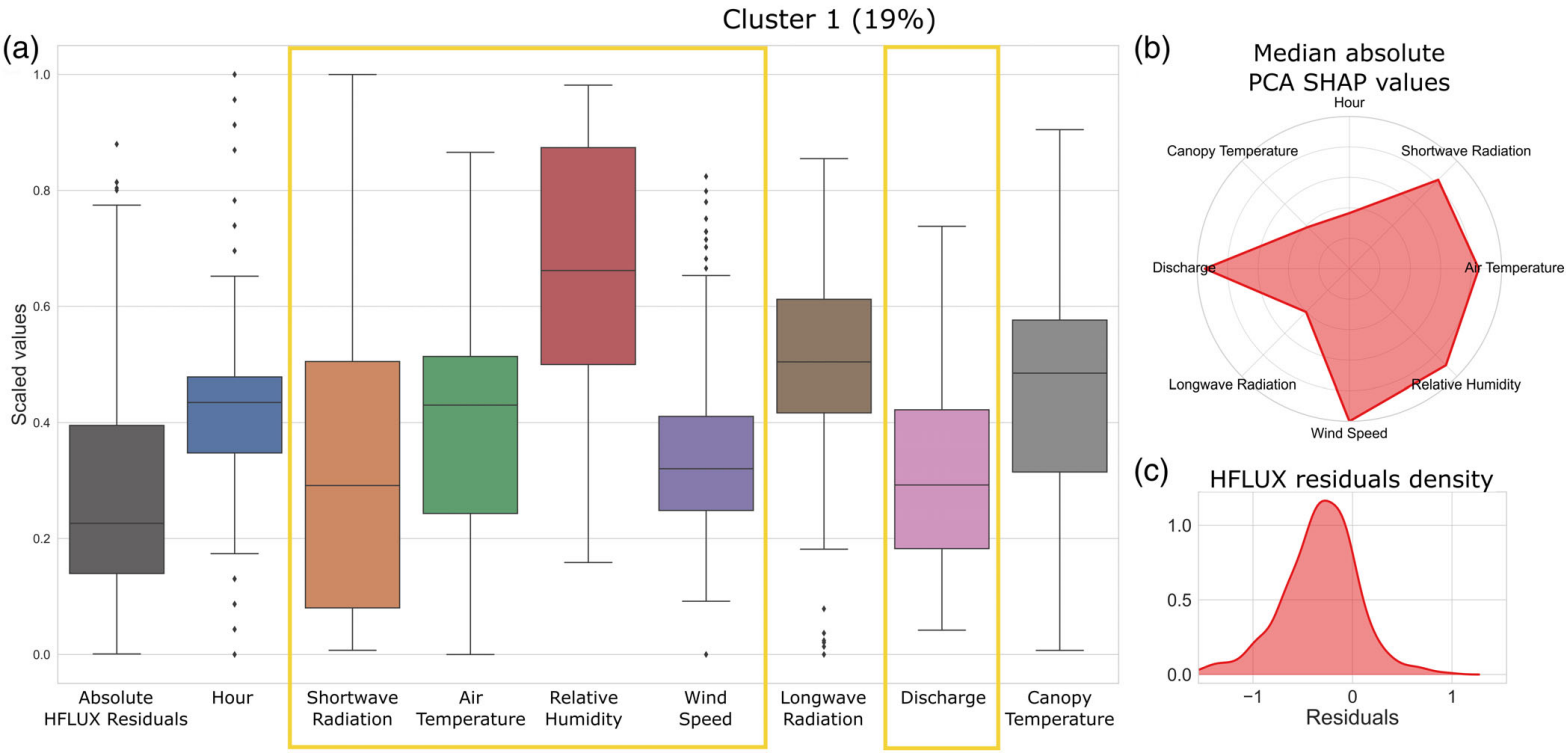
Properties of the first error cluster. (a) The boxplots of scaled values of the absolute HFLUX residuals and all variables of the cluster. Variables with large influence on cluster 1 errors are highlighted with yellow rectangles. The percentage value in the title shows the relative frequency of cluster 1 errors over the whole study time period. (b) The median absolute PCA SHAP values as an indicator of the importance of variables for this cluster formation. (c) The density of process‐based model residuals

The characteristics of cluster 1 are summarised in Figure 8a–c. Figure 8a shows the boxplots of standardised values of the absolute HFLUX residuals and all variables of the cluster. Figure 8b shows the median absolute PCA SHAP values as an indicator of the importance of variables for this cluster formation. For comparability, the scale of this figure type is consistent throughout all figures illustrating different error types. Figure 8c shows the counts of negative and positive process‐based model residuals, which correspond to under‐ and overestimation, respectively.

The results in Figure 8b shows that the errors of cluster 1 are strongly related to the variables discharge, shortwave radiation, air temperature, relative humidity and wind speed. They mainly occur in the late morning and are almost always positive, showing that cluster 1 errors mainly occur during warming periods of the stream, in which HFLUX is consistently predicting a more rapid increase in stream temperature than observed.

Since air temperature, shortwave radiation and relative humidity are highly correlated (shown in Figure 4), we can observe a similar importance for predicting cluster 1 errors in Figure 8b. However, canopy temperature does not seem to be as important, even though it is also highly correlated with these variables (mean correlation of 0.78).

**FIGURE 9:**
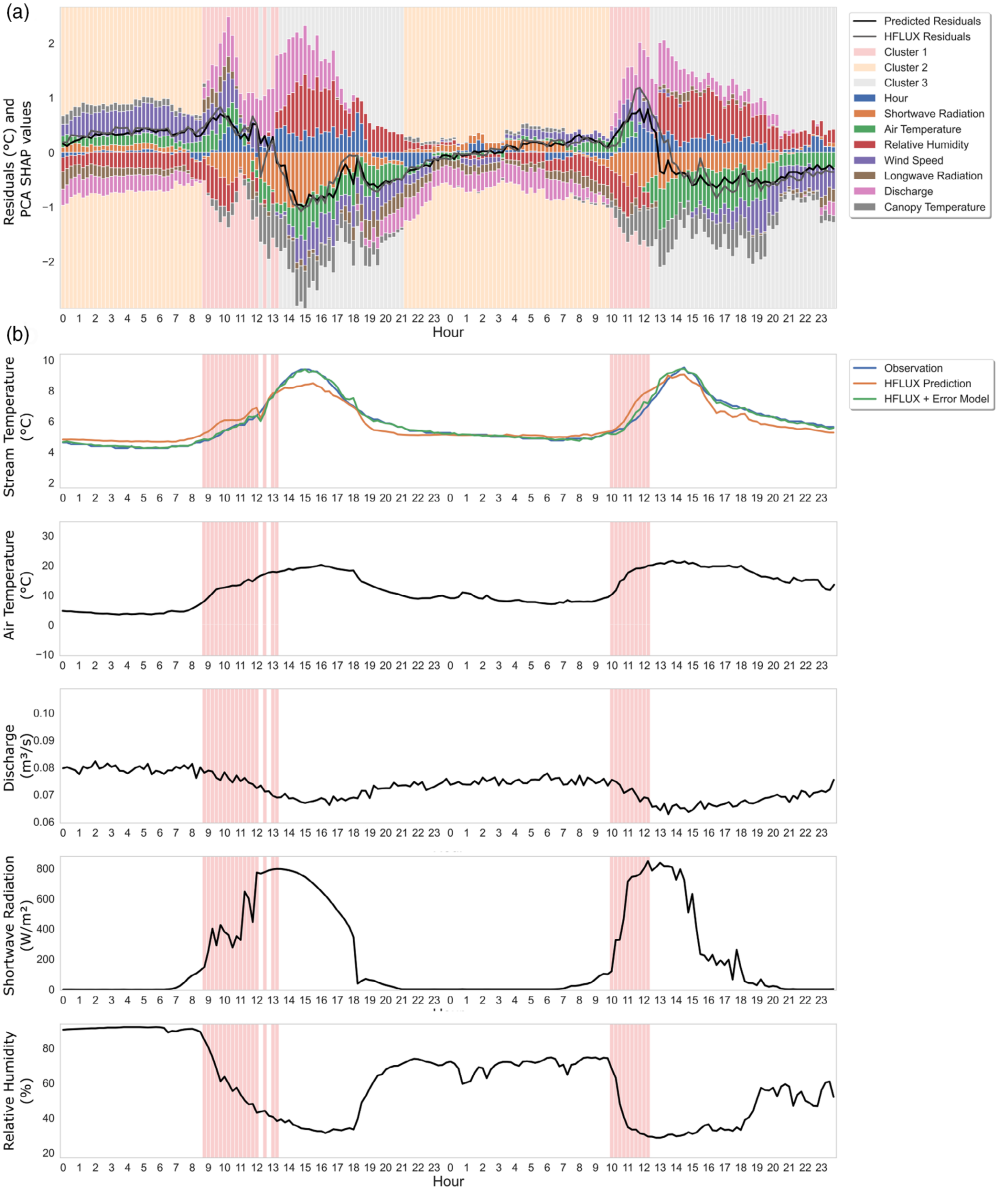
Cluster 1 example: 20–21 August 2019. (a) ML‐model predicted residuals, HFLUX model residuals, PCA SHAP values and background colours reflecting cluster affiliation. (b) time series of observed and predicted stream water temperature and variables with large influence on cluster 1 errors. Time periods of cluster 1 are highlighted

Figure 9a shows the PCA SHAP values and observed and predicted residuals for 20–21 August 2019, and Figure 9b shows the time series of the influencing variables for cluster 1 for the same time period. The yellow frames in Figure 9b highlight the occurrence times of cluster 1 errors. The PCA SHAP values and observed and predicted residuals for the full study period can be found in the Data S1 (supporting information S2). Cluster 1 errors always occur during times of decreasing relative humidity and discharge and increasing shortwave radiation and air temperature. Wind speed is increasing during cluster 1 errors in this example, but this does not apply to the full study period in general. For this stream HFLUX was generally relatively insensitive to changes in wind speed.

The PCA SHAP values in Figure 9a show that especially discharge, wind speed and air temperature are related to the overestimation, while relative humidity and shortwave radiation values result in PCA SHAP values that predict lower values. It is also noteworthy that PCA SHAP values for shortwave radiation and air temperature have different signs, even though these variables are highly correlated (0.64).

From these results we propose the following hypothesis for the error‐process relationship of cluster 1: These errors occur due to a combination of (1) measurement uncertainties during decreasing discharge, and (2) presumably faster warming of the meadow, in which the meteorological station is located, compared to the conditions in the river reach, for which HFLUX is applied to.

#### 3.3.2 Cluster 2: Vegetation‐emitted longwave radiation

**FIGURE 10:**
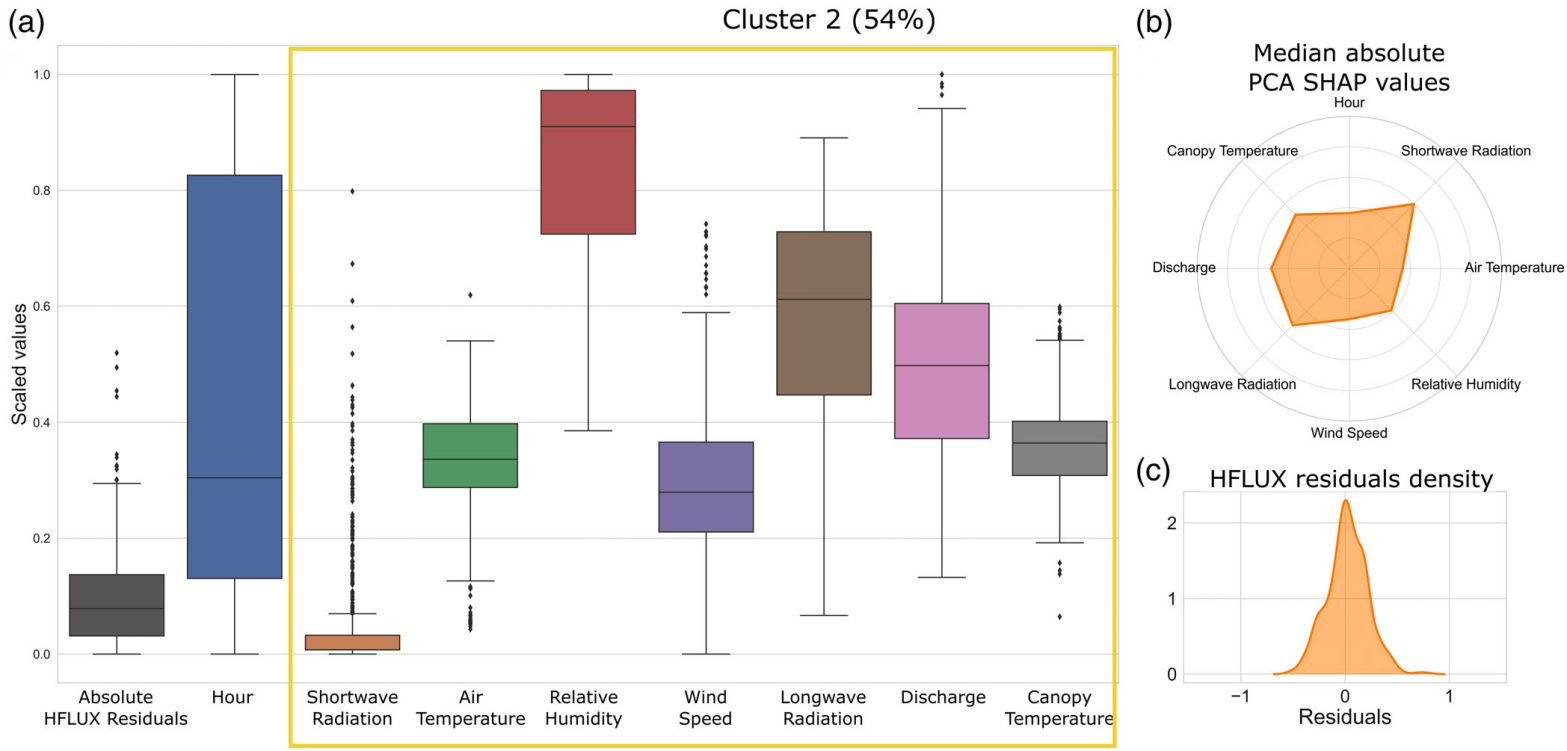
Properties of the second error cluster. (a) The boxplots of scaled values of the absolute HFLUX residuals and all variables of the cluster. Variables with large influence on cluster 2 errors are highlighted with yellow rectangles. The percentage value in the title shows the relative frequency of cluster 2 errors over the whole study time period. (b) The median absolute PCA SHAP values as an indicator of the importance of variables for this cluster formation. (c) The density of process‐based model residuals

The characteristics of cluster 2 are shown in Figure 10a–c. This cluster has the lowest residuals which occur during the night or on cloudy days, thus in times of low or no shortwave radiation input. Both under‐ and overestimation are present. Besides the low or absent shortwave radiation, Figure 10b shows that canopy temperature, longwave radiation, relative humidity and discharge are associated with the prediction of these errors. During cluster 2 error occurrence relative humidity is always quite high, longwave radiation tends to be higher than average and canopy temperature is generally lower than or equal to air temperature with a mean difference of −1.1°C.

**FIGURE 11:**
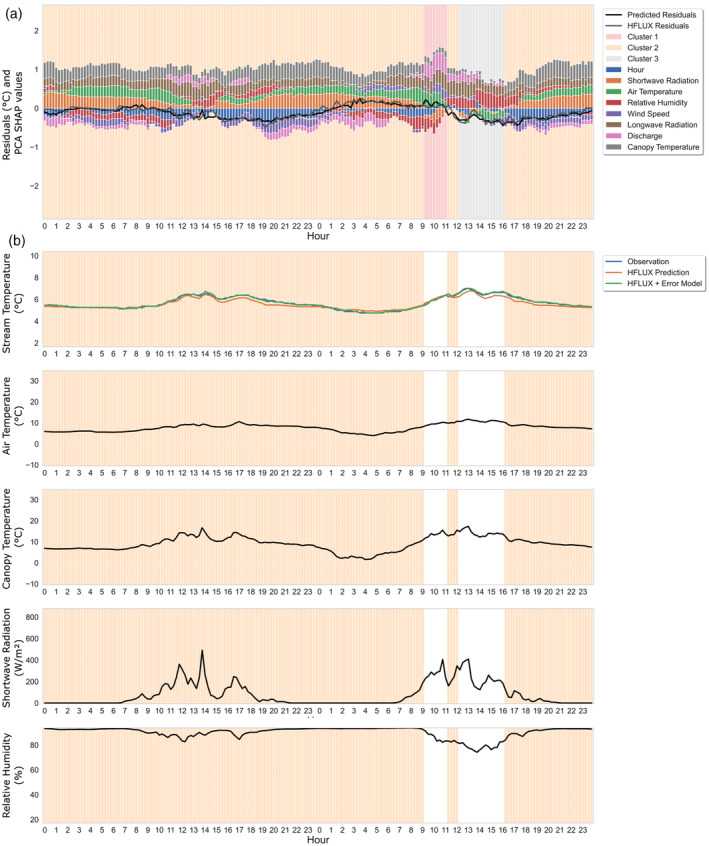
Cluster 2 example: 10–11 August, 2019. (a) ML‐model predicted residuals, HFLUX model residuals, PCA SHAP values and background colours reflecting cluster affiliation. (b) time series of observed and predicted stream water temperature and variables with large influence on cluster 2 errors. Time periods of cluster 2 are highlighted

Figure 11 shows an example time series for cluster 2 for 10–11 August 2019. During cluster 2 errors, air temperature and canopy temperature only increase slightly during the day and relative humidity is constantly above 90%. Shortwave radiation is low, with small peaks around 400 W/m^2^ around noon on both days. Canopy temperature, longwave radiation and air temperature show the largest PCA SHAP values in cluster 2 on these days. Shortwave radiation has large PCA SHAP values at night or early morning, but low PCA SHAP values during early afternoon, when high values are observed.

From these results we propose the following hypothesis for the error‐process relationship of cluster 2: Cluster 2 errors are mainly related to longwave radiation energy fluxes and to a smaller part to sensible and latent heat energy fluxes. The longwave radiation flux in HFLUX is estimated by incoming solar longwave radiation and vegetation‐emitted longwave radiation. Since HFLUX uses air temperature to estimate vegetation‐emitted longwave radiation, differences between these two will lead to over‐ and underestimation of this flux depending on the sign of the differences. The influence of relative humidity, air temperature and wind speed show also that some errors are associated with uncertainties in latent and sensible heat fluxes. This is most likely due to differences between the measurements made over the meadow compared to the conditions around the modelled stream reach.

#### 3.3.3 Cluster 3: Canopy temperature

**FIGURE 12:**
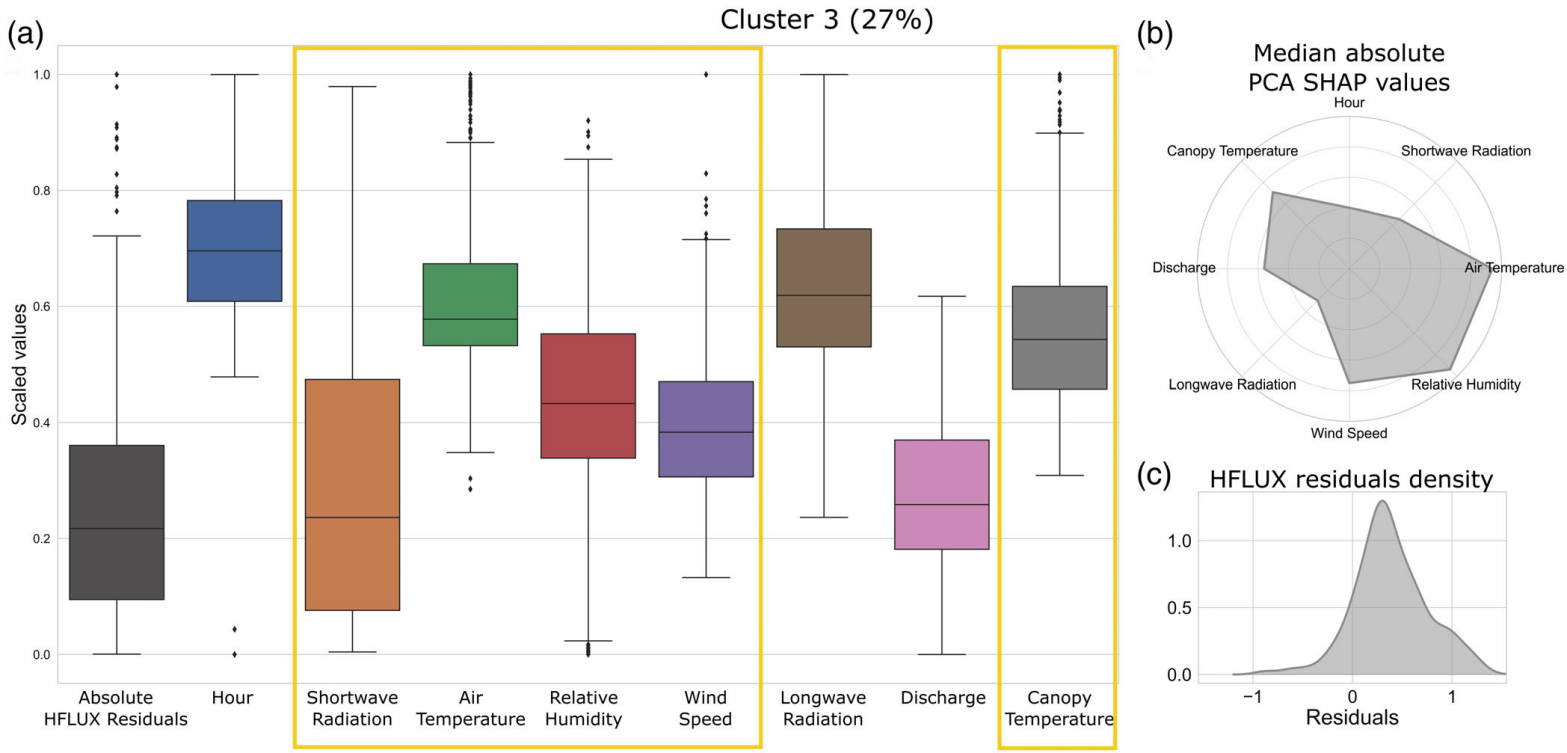
Properties of the third error cluster. (a) The boxplots of scaled values of the absolute HFLUX residuals and all variables of the cluster. Variables with large influence on cluster 2 errors are highlighted with yellow rectangles. The percentage value in the title shows the relative frequency of cluster 3 errors over the whole study time period. (b) The median absolute PCA SHAP values as an indicator of the importance of variables for this cluster formation. (c) The density of process‐based model residuals

The characteristics of cluster 3 are shown in Figure 12a–c. Cluster 3 errors are characterised by medium to high shortwave radiation, high air and canopy temperature and medium to low relative humidity. The absolute PCA SHAP values in Figure 12b show that these errors are mainly driven by shortwave radiation, air temperature, relative humidity, wind speed discharge and canopy temperature. They are mainly negative and occur in the afternoon, thus stream temperature is underestimated in times of high shortwave radiation.

**FIGURE 13:**
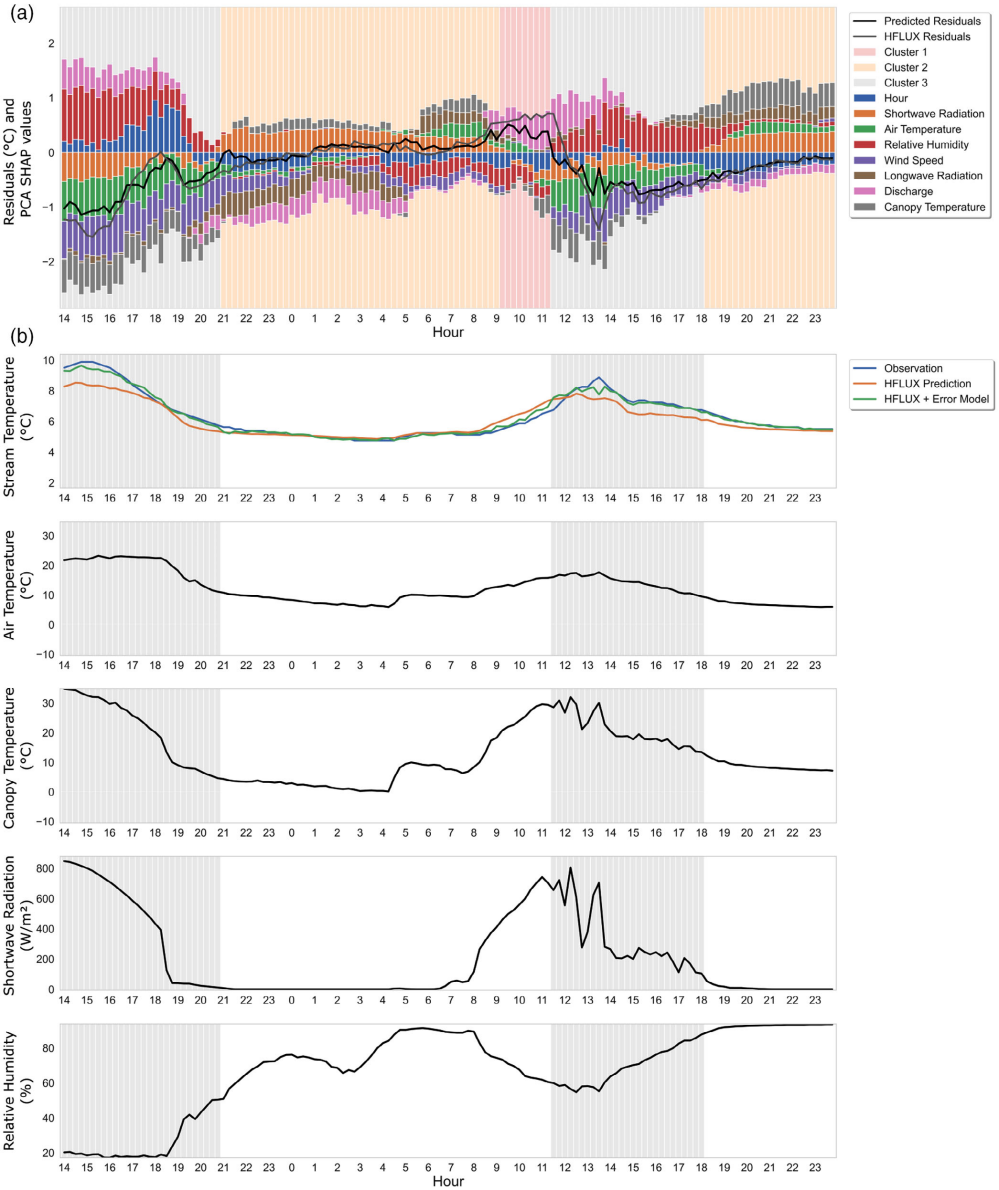
Cluster 3 example: 8–9 August 2019. (a) ML‐model predicted residuals, HFLUX model residuals, PCA SHAP values and background colours reflecting cluster affiliation. (b) time series of observed and predicted stream water temperature and variables with large influence on cluster 3 errors. Time periods of cluster 3 are highlighted

Figure 13 show an example time series for cluster 3 for 8–9 August, 2019. Cluster 3 errors always occur during times in which canopy temperature is much higher than air temperature and end when they are nearly equal again. They are characterised by low or increasing relative humidity. Shortwave radiation is usually high at the beginning but decreasing until zero at the end of each period.

From these results we propose the following hypothesis for the error‐process relationship of cluster 3: Cluster 3 errors mainly result from a combination of errors in shortwave radiation and longwave radiation. Due to the timing and importance of shortwave radiation we assume that the constant shading value estimated by HFLUX is too high in the time between 12:00 and 17:00. Since the shortwave radiation represents the largest energy input into the system, uncertainties in shading can result in large underestimations. In addition to shading related errors, it can also be assumed that a smaller part of the error is related to longwave radiation. During cluster 3 errors, measured canopy temperature is higher than air temperature (median difference 1.4°C, max difference 14.7°C), leading to an underestimation of the vegetation‐emitted longwave radiation.

### 3.4 Error hypotheses testing

**FIGURE 14:**
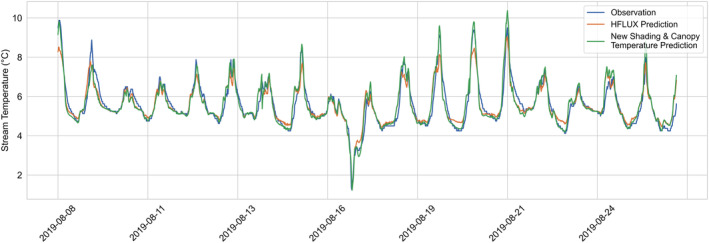
Comparison of observed and predicted time series of stream temperatures. Observed values are shown in blue, initial HFLUX predictions in orange and HFLUX predictions with new shading scheme and measured canopy temperatures as the basis for longwave radiation estimation are shown in green

Based on the proposed hypotheses we assume that a large part of the observed errors is related to two energy fluxes and their representation in the process‐based model: the shortwave radiation and longwave radiation energy fluxes. To test this assumption, we modified the HFLUX model to include a two‐point shading factor and used measured canopy temperature instead of air temperature to estimate vegetation‐emitted longwave radiation. The implemented two‐point shading included no further calibration, as it only modified the existing shading estimate to have temporal variability. This was done by linearly decreasing the existing shading factor estimate (that had spatial‐ but no temporal‐variability) to zero from 12:00 until 14:30 and then again linearly increase it back to the existing shading factor at 17:00. This choice was made based on experience from field work, where it was observed that the position of the sun around 14:30 was such that it shone along the length of the stream. The starting point at 12:00 was chosen since it is most often the starting time of cluster 3 errors and 17:00 was chosen as end point to make the two‐point shading symmetrical. Furthermore, we adapted HFLUX to use measured canopy temperature for estimating vegetation‐emitted longwave radiation. The resulting changes in HFLUX predictions are shown in Figure 14. While the prediction RMSE only improves slightly from 0.378°C to 0.348°C, visual inspection shows that some of the large errors during daily maximum stream temperature and during low stream temperatures at night are considerably reduced. Overall, it led to an RMSE reduction during night‐time (21:00–6:00) from 0.193°C to 0.149°C and in the afternoon (12:00–17:00) from 0.499°C to 0.428°C.

Uncertainties related to low and decreasing discharge as presumably occurring in cluster 1 errors, should be visible in the stage‐discharge rating curve of the gauging station. Therefore, we assessed the uncertainties of the rating curve of GS2 in the range of discharge values that occurred in August 2019. The rating curve is shown in the Data S1 (supporting information S4). It shows an increasing uncertainty in discharge estimation in times of low and declining water levels, since the spread in the observations in these periods around the rating curve is larger compared to other flow conditions.

## 4 DISCUSSION

This study introduces the LFM workflow and shows its application in a case study using the stream temperature model HFLUX. The LFM workflow enables the derivation of local explanations that are not influenced by correlated features and thus robustly reflect the relation of variables and model residuals. The results of the case study show three clusters of errors, which we assume to be mainly related to uncertainties in shading and vegetation‐emitted longwave radiation. We test these hypotheses with adjustments to HFLUX and its inputs without further calibration and could show that the errors related to these processes were reduced.

The LFM workflow is applicable to any process‐based model and will mainly differ in the type of selected ML‐model. However, the usage of the derived error‐process relationships will dependent on the type of application. For example, HFLUX could be implemented with the aim to estimate groundwater temperature. This can be achieved by calibrating and thus inferring groundwater temperature in HFLUX after measuring all other energy fluxes influencing stream water temperature. From the presented results we can assume that this would not be significantly influenced by the observed error groups as groundwater temperature does not influence daily peaks and pits were most of the model errors occur. If the aim would be to estimate the influence of vegetation shading on stream temperature to either assess planned or projected changes in vegetation, it would be necessary to adjust the model to include either calibrated or measured time‐varying shading factors. Therefore, this information can either be used to enhance trust in important processes or provide information on the potential areas of improvement to the model.

The results of the HFLUX case study also showed that some errors are easier to relate to processes (cluster 2 and 3 errors), while others are more difficult to interpret (cluster 1 errors). We found suitable hypotheses for all error clusters and could test the validity of two of them by adjusting the model without further calibration. To fully verify or falsify the cluster 1 error hypothesis evident in warming periods, further measurements would most likely be needed. However, the observed uncertainties in discharge estimation during low and declining water levels show that the first part of this hypothesis is valid. Regarding cluster 2 and 3 however, being able to show improvement in the expected time steps by simple model and model input adjustments, provides strong evidence for the proposed hypotheses.

The simple adjustment to the shading factor, which is a rough estimate based on the experience from field work in the catchment, is most likely not the solution which best fits the amount of shading that will be observed at every part of the stream. However, it shows the importance of temporal variability in shading when modelling the thermal system of a stream. In a study that focuses on an application related to shading, it would be necessary to include measurements to estimate shading along the stream at different times of the day, for example, with LiDAR (Loicq et al., 2018), hemispherical images (Garner et al., 2017), or a densiometer (Gravelle & Link, 2007).

The proposed PCA SHAP method results in explanations that also reflect feature dependence in the data. By using PCs of data as input for the ML‐model, the assumption of feature independence will always be true. Since this assumption is necessary for SHAP (Aas et al., 2021), we also know that the resulting SHAP values reflect an unbiased variable‐error relationship. While computing single variable PCA SHAP values from PCA loadings results in a loss of the SHAP local accuracy property, it allows for values that reflect unbiased explanations of all variables for a single prediction. Furthermore, the usage of multiple cluster methods and numbers of clusters in combination with a cluster quality metric, allows for objectivity and thus enhances trust in the derived clusters. Iterative procedures like k‐means can still produce slightly different results any time they are applied, but this will not change the general cluster properties and thus their interpretation.

Model errors contain valuable information that should be used to further understand physical processes and process‐based models. By applying the presented workflow, it should be possible to derive data‐based hypothesis on error generation for any type of model. Especially the PCA extension for SHAP values should make it applicable to any dataset, where correlated features might heavily influence interpretability. This type of data driven approach allows using objective data driven methods to be used to further understand and develop process‐based models. ML‐models have already been widely applied to estimate model residuals of process‐based models to improve predictions (e.g., Konapala et al., 2020; Mekonnen et al., 2015; Wu et al., 2019; Xu et al., 2017). At the same time, process‐based models are especially important for tasks in which ML‐models are not applicable, that is, understanding processes and/or predicting the results of changes in the system. Thus, only improving prediction will not assist these types of application. Recent work by Bennett and Nijssen (2021) shows a coupled process‐based model and neural network approach with an explainable machine‐learning method to further understand a specific process. Similarly, the LFM workflow was developed to enhance and improve understanding of process‐based model applications for any type of model.

Besides these applications, the improvement of model performance by an error ML‐model can potentially be seen as a quantity of model limitation or error. It represents the information that is still contained in the data, which could potentially be used to improve the model. Similarly, it is also interesting that the error ML‐model does not achieve perfection. The difference to a RMSE of zero could be seen as a quantification of other sources of uncertainties, such as uncertainties in the input data, or additional non‐known controlling variables. These quantities can potentially lead to further evidence for model development and process understanding.

This study has potential limitations. Applying the LFM workflow needs in‐depth knowledge of the physical system and its processes, the input ML‐model input data, and to some extent, machine‐learning prediction models. This can be a potentially limiting factor, if expertise in one of those areas is not available. To mitigate this limitation, we provide the python code and data to reproduce the presented results, thereby providing a blueprint and template for other applications. Another limitation is the validity of the resulting hypotheses on error generation. Without additional tests or measurements, these hypotheses cannot be verified or falsified with certainty. In simple cases, the resulting hypothesis will be sufficient and obviously reasonable, in other cases this could lead to wrong assumptions.

## 5 CONCLUSIONS

In this work we found that learning from model errors is possible and a worthwhile pursuit when applying process‐based models for gaining knowledge or decision making. The developed approach of estimating explanations for ML‐models that are not influenced by feature dependence, is convenient and can also be used for other ML applications in hydrology. In a next step it would be interesting to apply the procedure to more complex models with a specific research question related to either decision making or process understanding. It would also be interesting to investigate if the error‐process relationships and the error model can be transferred to another river or river‐branch.

## Supporting information


**Data S1.** Choosing a ML‐model for residual prediction.
**Data S2.** PCA SHAP values, HFLUX predictions and ML‐model prediction plots.
**Data S3.** Timeseries of variables for 8–16 August 2019.
**Data S4.** Stage‐discharge rating curve for GS2.

## Data Availability

The Python code used to generate all results for this publication can be found in Feigl (2021). The hydro‐meteorological data and HFLUX inputs/outputs used in this study are available from Feigl, Roesky, et al. (2021).

## APPENDIX A

Optimised XGBoost hyperparameters and their chosen lower and upper bounds: n_estimators [50, 5000], learning_rate [0, 1], max_depth [3, 20], gamma [0.01, 5], min_child_weight [0, 10], max_delta_step [0, 5], subsample [0.5, 1.0], colsample_bytree [0.3, 10], scale_pos_weight [1.2, 5.0], reg_alpha [4, 10], reg_lambda [1, 10].

## References

[hyp14515-bib-0001] Aas, K. , Jullum, M. , & Løland, A. (2021). Explaining individual predictions when features are dependent: More accurate approximations to Shapley values. Artificial Intelligence, 298, 103502. 10.1016/j.artint.2021.103502

[hyp14515-bib-0002] Babovic, V. , Caňizares, R. , Jensen, H. R. , & Klinting, A. (2001). Neural networks as routine for error updating of numerical models. Journal of Hydraulic Engineering, 127(3), 181–193. 10.1061/(asce)0733-9429(2001)127:3(181)

[hyp14515-bib-0003] Baehrens, D. , Harmeling, S. , Kawanabe, M. , Hansen Khansen, K. , & Edward Rasmussen, C. (2010). How to explain individual classification decisions Timon Schroeter * Klaus‐Robert Muller. Journal of Machine Learning Research, 11, 1803–1831.

[hyp14515-bib-0004] Bennett, A. , & Nijssen, B. (2021). Explainable AI uncovers how neural networks learn to regionalize in simulations of turbulent heat fluxes at FluxNet sites. Earth and Space Science Open Archive, 23.

[hyp14515-bib-0005] Beven, K. (1989). Changing ideas in hydrology‐the case of physically‐based models. Journal of Hydrology, 105(1–2), 157–172. 10.1016/0022-1694(89)90101-7

[hyp14515-bib-0006] Beven, K. (2016). Facets of uncertainty: Epistemic uncertainty, non‐stationarity, likelihood, hypothesis testing, and communication. Hydrological Sciences Journal, 61(9), 1652–1665. 10.1080/02626667.2015.1031761

[hyp14515-bib-0007] Blöschl, G. , & Sivapalan, M. (1995). Scale issues in hydrological modelling: A review. Hydrological Processes, 9(3–4), 251–290. 10.1002/HYP.3360090305

[hyp14515-bib-0008] Chen, T. , & Guestrin, C. (2016). *XGBoost: A scalable tree boosting system*. Proceedings of the ACM SIGKDD International Conference on Knowledge Discovery and Data Mining, 13‐17‐Augu(8), pp. 785–794. 10.1145/2939672.2939785

[hyp14515-bib-0009] Davies, D. L. , & Bouldin, D. W. (1979). A cluster separation measure. IEEE Transactions on Pattern Analysis and Machine Intelligence, 1(2), 224–227. 10.1109/TPAMI.1979.4766909 21868852

[hyp14515-bib-0010] Demissie, Y. K. , Valocchi, A. J. , Minsker, B. S. , & Bailey, B. A. (2009). Integrating a calibrated groundwater flow model with error‐correcting data‐driven models to improve predictions. Journal of Hydrology, 364(3–4), 257–271. 10.1016/j.jhydrol.2008.11.007

[hyp14515-bib-0011] Dingman, S. L. (1994). Physical hydrology. Macmillan Pub. Co.

[hyp14515-bib-0012] Dunn, J. C. (2008). A fuzzy relative of the ISODATA process and its use in detecting compact well‐separated clusters, 3(3), 32–57. 10.1080/01969727308546046

[hyp14515-bib-0013] Feigl, M. (2021). *Learning‐from‐mistakes: Code v1.0*. Zenodo. 10.5281/zenodo.5764667

[hyp14515-bib-0014] Feigl, M. , Lebiedzinski, K. , Herrnegger, M. , & Schulz, K. (2021). Machine‐learning methods for stream water temperature prediction. Hydrology and Earth System Sciences, 25(5), 2951–2977. 10.5194/HESS-25-2951-2021

[hyp14515-bib-0015] Feigl, M. , Roesky, B. , Herrnegger, M. , Schulz, K. , & Hayashi, M. (2021). Data for “learning from mistakes‐assessing the performance and uncertainty in process‐based models.” 10.5281/ZENODO.5185399 PMC9306826 35910683

[hyp14515-bib-0016] Garner, G. , Malcolm, I. A. , Sadler, J. P. , & Hannah, D. M. (2017). The role of riparian vegetation density, channel orientation and water velocity in determining river temperature dynamics. Journal of Hydrology, 553, 471–485. 10.1016/J.JHYDROL.2017.03.024

[hyp14515-bib-0017] Glose, A. M. , Lautz, L. K. , & Baker, E. A. (2017). Stream heat budget modeling with HFLUX: Model development, evaluation, and applications across contrasting sites and seasons. Environmental Modelling and Software, 92, 213–228. 10.1016/j.envsoft.2017.02.021

[hyp14515-bib-0018] Gravelle, J. A. , & Link, T. E. (2007). Influence of timber harvesting on headwater peak stream temperatures in a northern Idaho watershed. Forest Science, 53(2), 189–205. 10.1093/FORESTSCIENCE/53.2.189

[hyp14515-bib-0019] Hoeffding, W. (1948). A non‐parametric test of independence. The Annals of Mathematical Statistics, 19, 546–557.

[hyp14515-bib-0020] Jemberie, A. (2004). Information theory and artificial intelligence to manage uncertainty in hydrodynamic and hydrological models. CRC Press.

[hyp14515-bib-0021] Jolliffe, I. T. (2002). Principal components in regression analysis. In Principal component analysis (pp. 167–198). Springer.

[hyp14515-bib-0022] Kirchner, J. W. (2006). Getting the right answers for the right reasons: Linking measurements, analyses, and models to advance the science of hydrology. Water Resources Research, 42(3), W03S04. 10.1029/2005WR004362

[hyp14515-bib-0023] Klemeš, V. (1986). Dilettantism in hydrology: Transition or destiny? Water Resources Research, 22(9S), 177S–188S. 10.1029/WR022I09SP0177S

[hyp14515-bib-0024] Konapala, G. , Kao, S. C. , Painter, S. L. , & Lu, D. (2020). Machine learning assisted hybrid models can improve streamflow simulation in diverse catchments across the conterminous US. Environmental Research Letters, 15(10), 104022. 10.1088/1748-9326/aba927

[hyp14515-bib-0025] Kushner, H. J. (1964). A new method of locating the maximum point of an arbitrary multipeak curve in the presence of noise. Journal of Fluids Engineering, Transactions of the ASME, 86(1), 97–106. 10.1115/1.3653121

[hyp14515-bib-0026] Loicq, P. , Moatar, F. , Jullian, Y. , Dugdale, S. J. , & Hannah, D. M. (2018). Improving representation of riparian vegetation shading in a regional stream temperature model using LiDAR data. Science of the Total Environment, 624, 480–490. 10.1016/J.SCITOTENV.2017.12.129 29268220

[hyp14515-bib-0027] Lundberg, S. M. , Allen, P. G. , & Lee, S.‐I. (2017). A unified approach to interpreting model predictions. In Advances in neural information processing systems (Vol. 30). Curran Associates, Inc.

[hyp14515-bib-0028] Lundberg, S. M. , Erion, G. , Chen, H. , DeGrave, A. , Prutkin, J. M. , Nair, B. , Katz, R. , Himmelfarb, J. , Bansal, N. , & Lee, S.‐I. (2020). From local explanations to global understanding with explainable AI for trees. Nature Machine Intelligence, 2(1), 56–67. 10.1038/s42256-019-0138-9 PMC7326367 32607472

[hyp14515-bib-0029] MacQueen, J. (1967). Some methods for classification and analysis of multivariate observations. Proceedings of the Fifth Berkeley Symposium on Mathematical Statistics and Probability, 1, 281–296.

[hyp14515-bib-0030] Mekonnen, B. A. , Nazemi, A. , Mazurek, K. A. , Elshorbagy, A. , & Putz, G. (2015). Hybrid modelling approach to prairie hydrology: Fusing data‐driven and process‐based hydrological models, 60(9), 1473–1489. 10.1080/02626667.2014.935778

[hyp14515-bib-0031] Močkus, J. (1989). Bayesian approach to global optimization (Vol. 37). Springer. 10.1007/978-94-009-0909-0

[hyp14515-bib-0032] Molnar, C. (2019). Interpretable machine learning. https://christophm.github.io/interpretable-ml-book/

[hyp14515-bib-0033] Molnar, C. , Casalicchio, G. , & Bischl, B. (2020). Interpretable machine learning‐a brief history, state‐of‐the‐art and challenges. Communications in Computer and Information Science, 1323, 417–431. 10.1007/978-3-030-65965-3_28

[hyp14515-bib-0034] Moore, R. D. , Sutherland, P. , Gomi, T. , & Dhakal, A. (2005). Thermal regime of a headwater stream within a clear‐cut, coastal British Columbia, Canada. Hydrological Processes, 19(13), 2591–2608. 10.1002/HYP.5733

[hyp14515-bib-0035] Pearson, K. (1901). LIII. On lines and planes of closest fit to systems of points in space. The London, Edinburgh, and Dublin Philosophical Magazine and Journal of Science, 2(11), 559–572. 10.1080/14786440109462720

[hyp14515-bib-0036] Reshef, D. N. , Reshef, Y. A. , Finucane, H. K. , Grossman, S. R. , McVean, G. , Turnbaugh, P. J. , Lander, E. S. , Mitzenmacher, M. , & Sabeti, P. C. (2011). Detecting novel associations in large data sets. Science, 334(6062), 1518–1524. 10.1126/science.1205438 22174245 PMC3325791

[hyp14515-bib-0037] Roesky, B. , & Hayashi, M. (2022). Effects of lake‐groundwater interaction on the thermal regime of a sub‐alpine headwater stream. Hydrological Processes, e14501. 10.1002/hyp.14501

[hyp14515-bib-0038] Rousseeuw, P. J. (1987). Silhouettes: A graphical aid to the interpretation and validation of cluster analysis. Journal of Computational and Applied Mathematics, 20, 53–65. 10.1016/0377-0427(87)90125-7

[hyp14515-bib-0039] Shapley, L. S. (1953). A value for n‐person games. Contributions to the Theory of Games, 2(28), 307–317.

[hyp14515-bib-0040] Snoek, J. , Larochelle, H. , & Adams, R. P. (2012). Practical Bayesian optimization of machine learning algorithms. Advances in Neural Information Processing Systems, 4, 2951–2959.

[hyp14515-bib-0041] Ward, J. H. (1963). Hierarchical grouping to optimize an objective function. Journal of the American Statistical Association, 58(301), 236–244. 10.1080/01621459.1963.10500845

[hyp14515-bib-0042] Wu, R. , Yang, L. , Chen, C. , Ahmad, S. , Dascalu, S. M. , & Harris, F. C. (2019). MELPF version 1: Modeling error learning based post‐processor framework for hydrologic models accuracy improvement. Geoscientific Model Development, 12, 4115–4131. 10.5194/gmd-12-4115-2019

[hyp14515-bib-0043] Xu, T. , Valocchi, A. J. , Choi, J. , & Amir, E. (2014). Use of machine learning methods to reduce predictive error of groundwater models. Groundwater, 52(3), 448–460. 10.1111/gwat.12061 23647322

[hyp14515-bib-0044] Xu, T. , Valocchi, A. J. , Ye, M. , & Liang, F. (2017). Quantifying model structural error: Efficient Bayesian calibration of a regional groundwater flow model using surrogates and a data‐driven error model. Water Resources Research, 53(5), 4084–4105. 10.1002/2016WR019831

[hyp14515-bib-0045] Young, C. C. , Liu, W. C. , & Wu, M. C. (2017). A physically based and machine learning hybrid approach for accurate rainfall‐runoff modeling during extreme typhoon events. Applied Soft Computing Journal, 53, 205–216. 10.1016/j.asoc.2016.12.052

