# Supplementary material for: Learning from mistakes—Assessing the performance and uncertainty in process‐based models
Source: Hydrol Process. 2022 Feb 24;36(2):e14515. doi: 10.1002/hyp.14515 (PMC9306826; doi:10.1002/hyp.14515)

**Supporting information**

**S1. Choosing a ML-model for residual prediction**

Setting up an adequate ml-model for residual prediction is arguably the most challenging part of this workflow but will in most cases be solvable with ready-to-use models from widely used ml-libraries. Generally, any model that can solve a regression type problem can be used for model residual prediction. However, most process-based model output and inputs will be either time series, or in more complex cases maps of time series, i.e. spatio-temporal distributions of a variable. Therefore, certain models will perform better than others. For time series prediction where long-term dependencies can be assumed as not relevant (Feigl et al., 2021), models like Random Forests, XGBoost (Chen & Guestrin, 2016), LightGBM (Ke et al., 2017), or Feedforward neural networks in combination with lagged inputs should be reasonable choices. In cases where long-term dependencies might be relevant, models like LSTMs (Hochreiter & Schmidhuber, 1997), GRUs (Cho et al., 2014), TCNs (Bai et al., 2018), Transformers (Vaswani et al., 2017) or CNNs (LeCun et al., 1989) are better choices, as they were originally made to work with sequences of inputs.

Predicting maps of time series is more challenging and can either be solved by training a model that predicts a time series for a single grid cell, or a model that predicts the full map for each time step. When predicting the residuals for each individual grid cell, the problem would be equal to the time series prediction problem and thus all models mentioned above would be applicable. Predicting full maps of residuals can be achieved with CNNs, or CNNs in combination with LSTMs, Transformers or Feedforward neural networks.

Bai, S., Kolter, J. Z., & Koltun, V. (2018). An Empirical Evaluation of Generic Convolutional and Recurrent Networks for Sequence Modeling. *ArXiv*. http://arxiv.org/abs/1803.01271

Chen, T., & Guestrin, C. (2016). XGBoost: A scalable tree boosting system. *Proceedings of the ACM SIGKDD International Conference on Knowledge Discovery and Data Mining*, *13*-*17*-*Augu*(8), 785–794. https://doi.org/10.1145/2939672.2939785

Cho, K., Van Merriënboer, B., Gulcehre, C., Bahdanau, D., Bougares, F., Schwenk, H., & Bengio, Y. (2014). Learning phrase representations using RNN encoder-decoder for statistical machine translation. *EMNLP 2014 - 2014 Conference on Empirical Methods in Natural Language Processing, Proceedings of the Conference*, 1724–1734. https://doi.org/10.3115/v1/d14-1179

Feigl, M., Lebiedzinski, K., Herrnegger, M., & Schulz, K. (2021). Machine-learning methods for stream water temperature prediction. *Hydrology and Earth System Sciences*, *25*(5), 2951–2977. https://doi.org/10.5194/HESS-25-2951-2021

Hochreiter, S., & Schmidhuber, J. (1997). Long Short-Term Memory. *Neural Computation*, *9*(8), 1735–1780. https://doi.org/10.1162/neco.1997.9.8.1735

Ke, G., Meng, Q., Finley, T., Wang, T., Chen, W., Ma, W., Ye, Q., & Liu, T.-Y. (2017). LightGBM: A Highly Efficient Gradient Boosting Decision Tree. *Advances in Neural Information Processing Systems*, *30*. https://github.com/Microsoft/LightGBM.

LeCun, Y., Boser, B., Denker, J. S., Henderson, D., Howard, R. E., Hubbard, W., & Jackel, L. D. (1989). Backpropagation Applied to Handwritten Zip Code Recognition. *Neural Computation*, *1*(4), 541–551. https://doi.org/10.1162/NECO.1989.1.4.541

Vaswani, A., Shazeer, N., Parmar, N., Uszkoreit, J., Jones, L., Gomez, A. N., Kaiser, L., & Polosukhin, I. (2017). Attention Is All You Need. *Advances in Neural Information Processing Systems*, *2017*-*December*, 5999–6009. https://arxiv.org/abs/1706.03762v5

**S2. PCA SHAP values, HFLUX predictions and ml-model prediction plots**


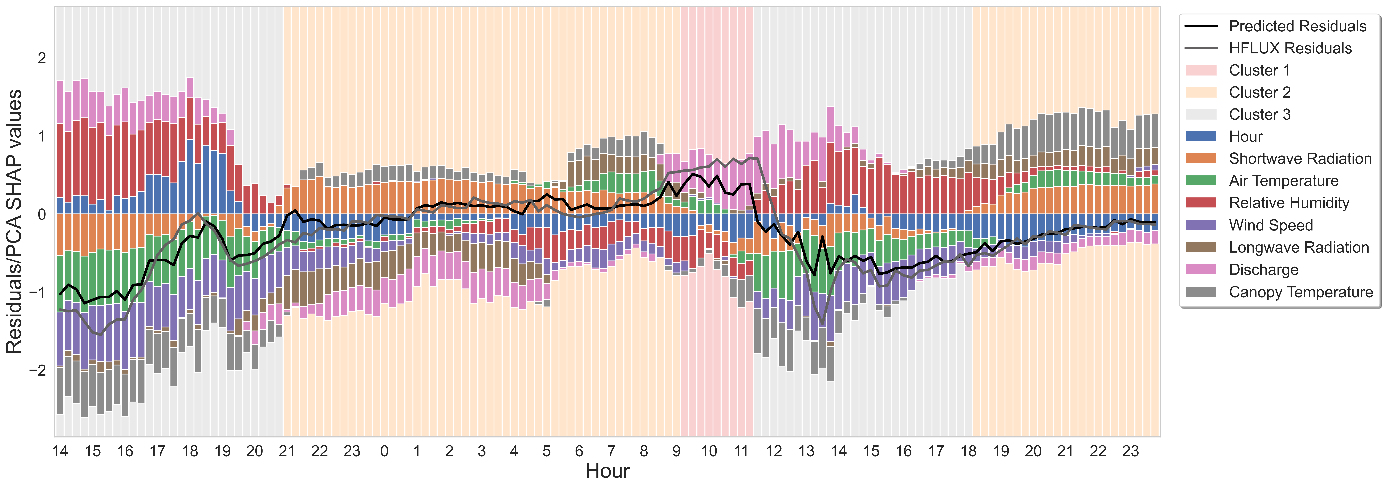


S2. 1 August 8-9, 2019: Ml-model predicted residuals, HFLUX model residuals, PCA SHAP values and background colours reflecting cluster affiliation


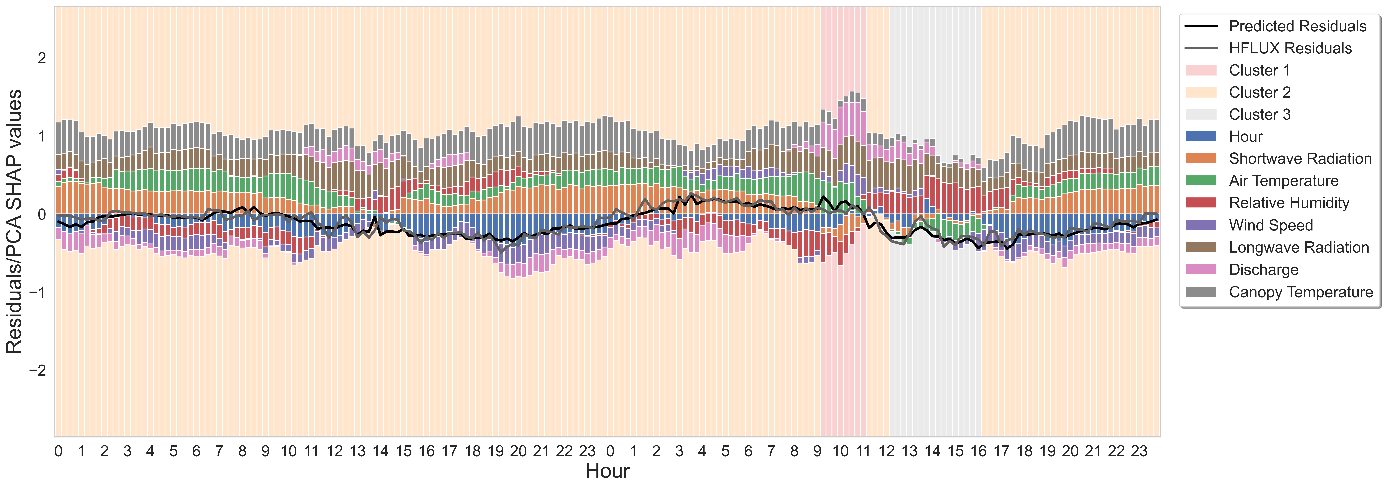


S2. 2 August 10-11, 2019: Ml-model predicted residuals, HFLUX model residuals, PCA SHAP values and background colours reflecting cluster affiliation


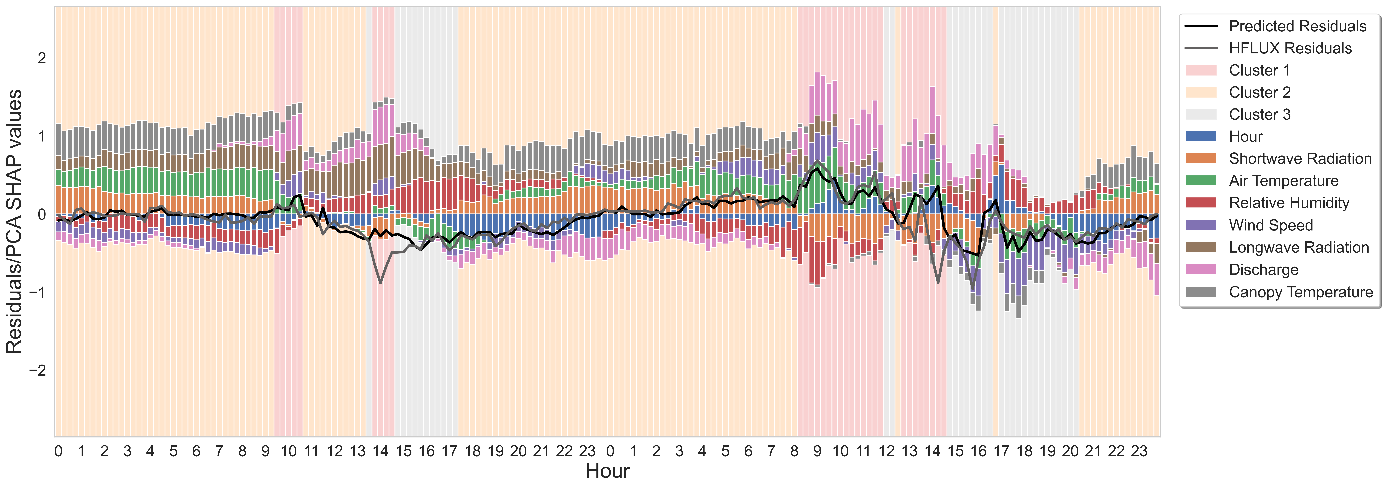


S2. 3 August 12-13, 2019: Ml-model predicted residuals, HFLUX model residuals, PCA SHAP values and background colours reflecting cluster affiliation


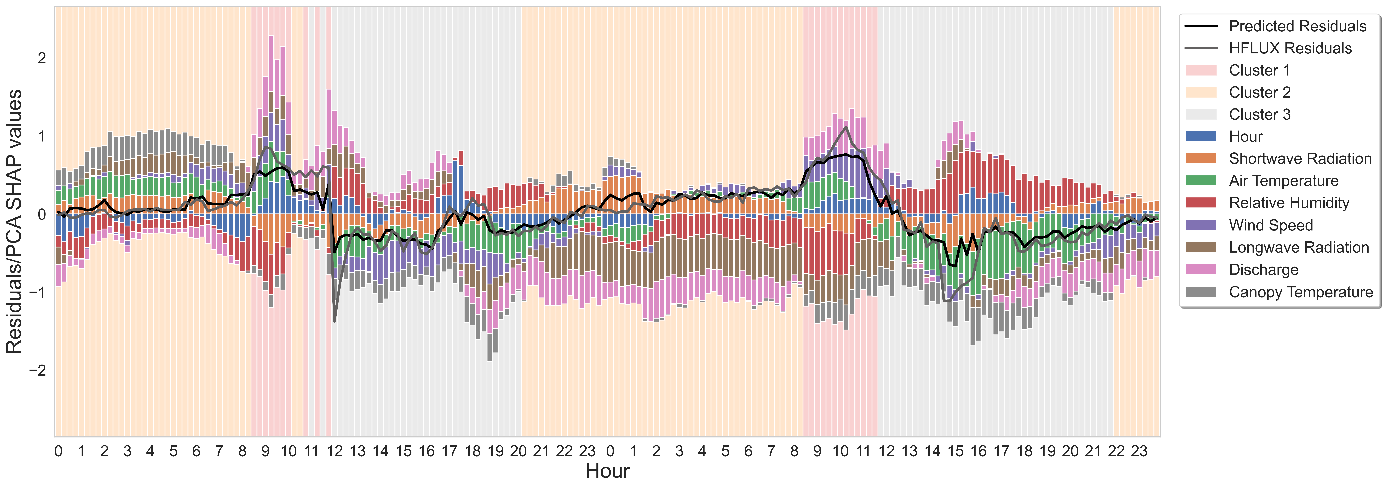


S2. 4 August 14-15, 2019: Ml-model predicted residuals, HFLUX model residuals, PCA SHAP values and background colours reflecting cluster affiliation


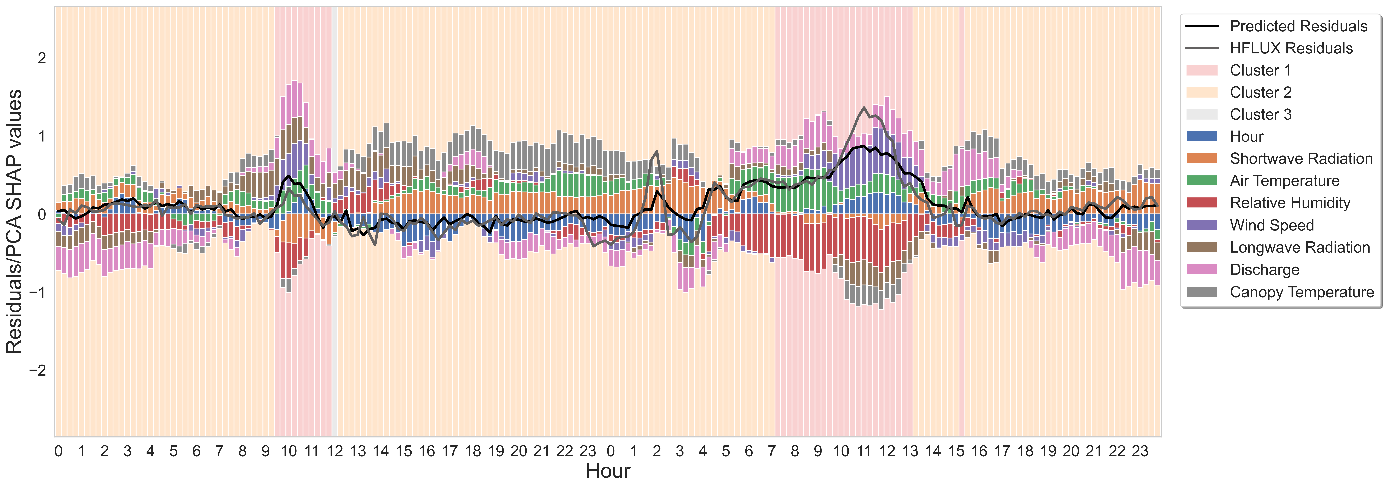


S2. 5 August 16-17, 2019: Ml-model predicted residuals, HFLUX model residuals, PCA SHAP values and background colours reflecting cluster affiliation


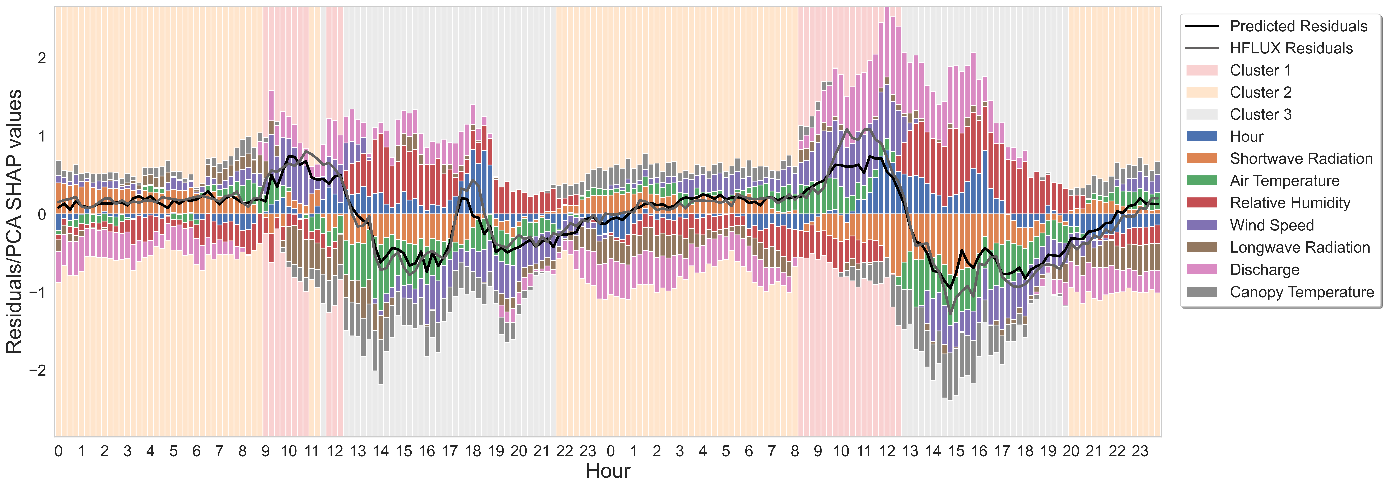


S2. 6 August 18-19, 2019: Ml-model predicted residuals, HFLUX model residuals, PCA SHAP values and background colours reflecting cluster affiliation


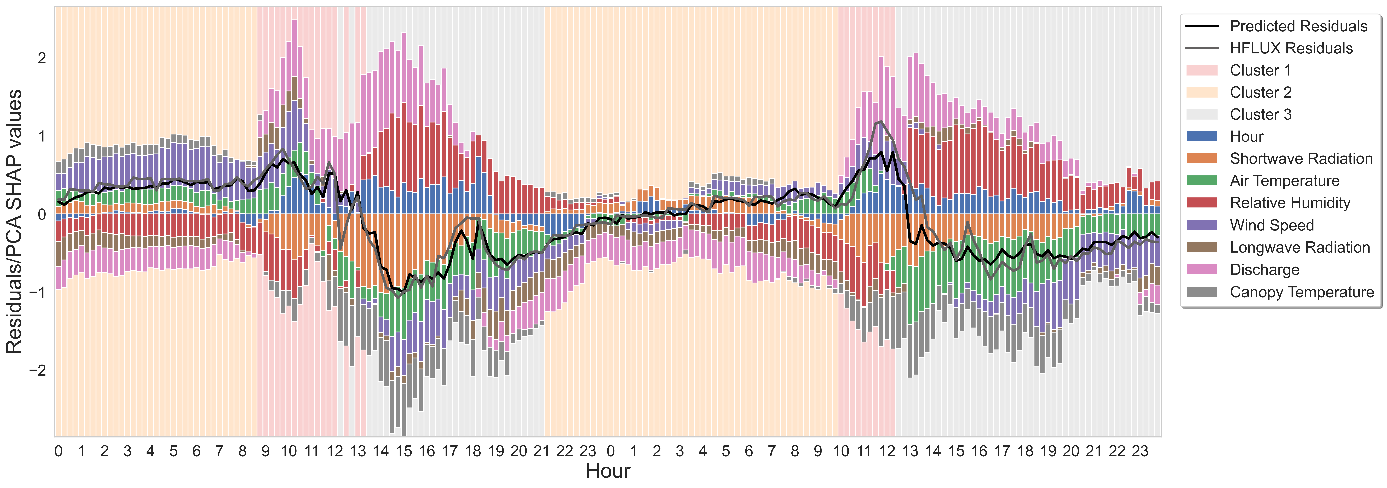


S2. 7 August 20-21, 2019: Ml-model predicted residuals, HFLUX model residuals, PCA SHAP values and background colours reflecting cluster affiliation


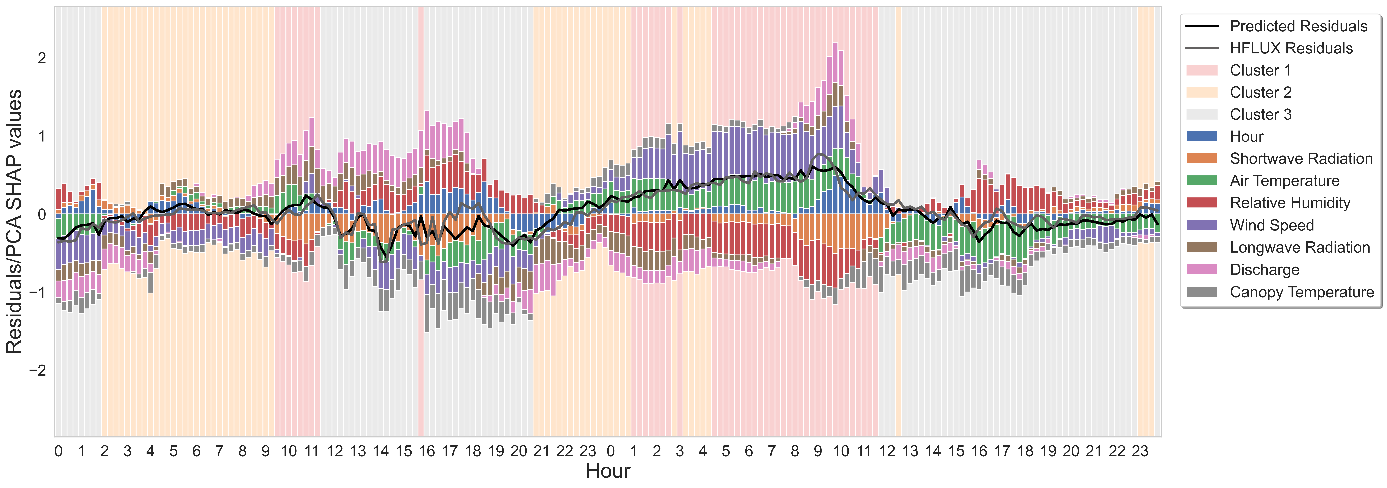


S2. 8 August 22-23, 2019: Ml-model predicted residuals, HFLUX model residuals, PCA SHAP values and background colours reflecting cluster affiliation


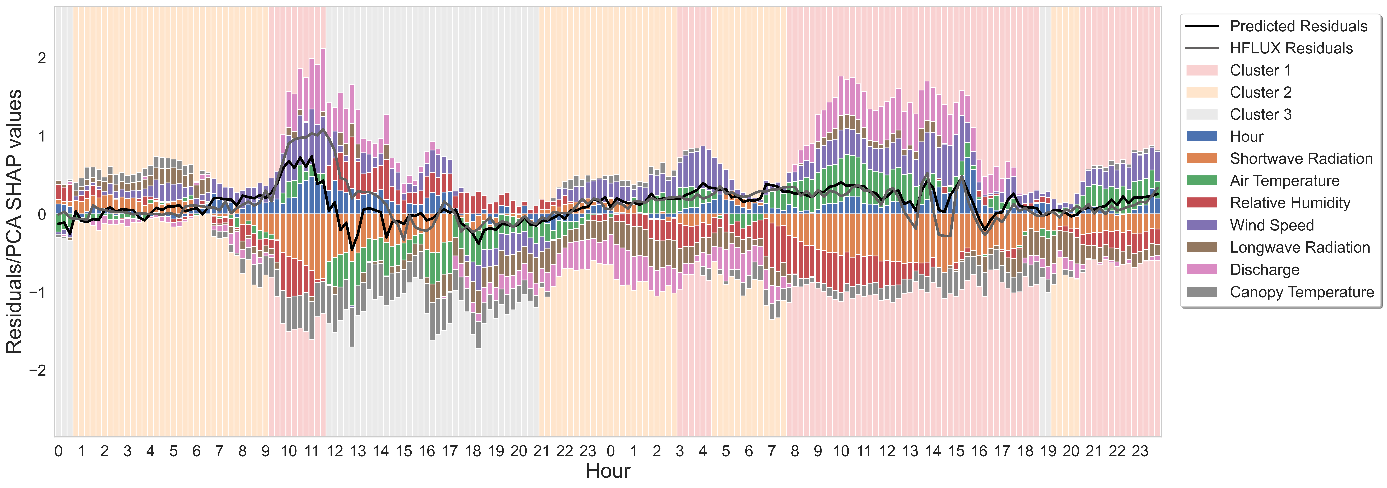


S2. 9 August 24-25, 2019: Ml-model predicted residuals, HFLUX model residuals, PCA SHAP values and background colours reflecting cluster affiliation


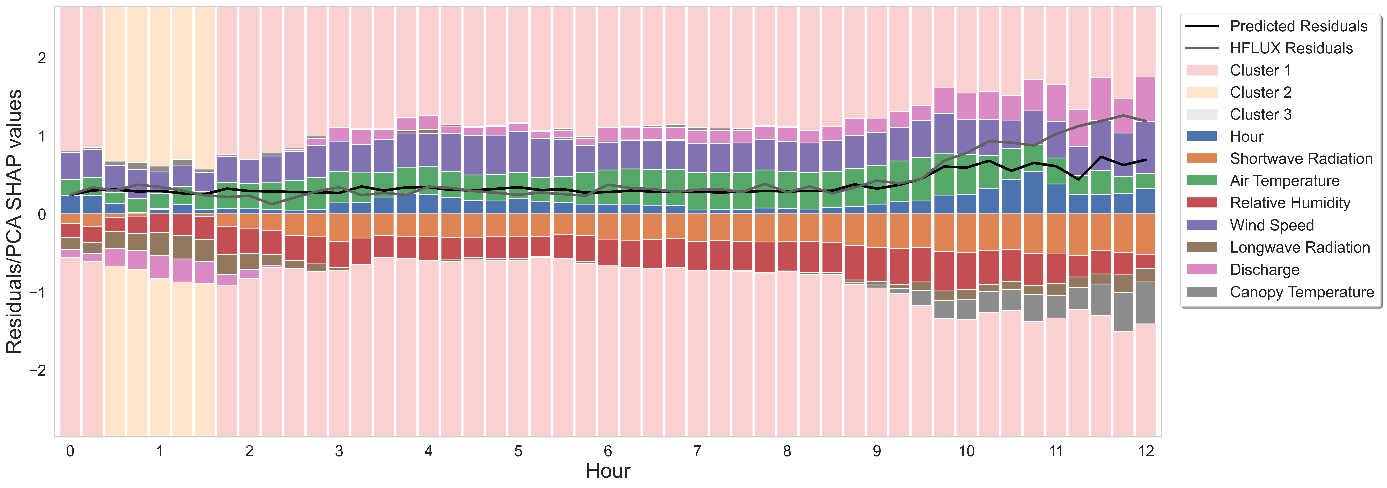


S2. 10 August 26, 2019: Ml-model predicted residuals, HFLUX model residuals, PCA SHAP values and background colours reflecting cluster affiliation

**S3. Timeseries of variables for August 8-16, 2019**


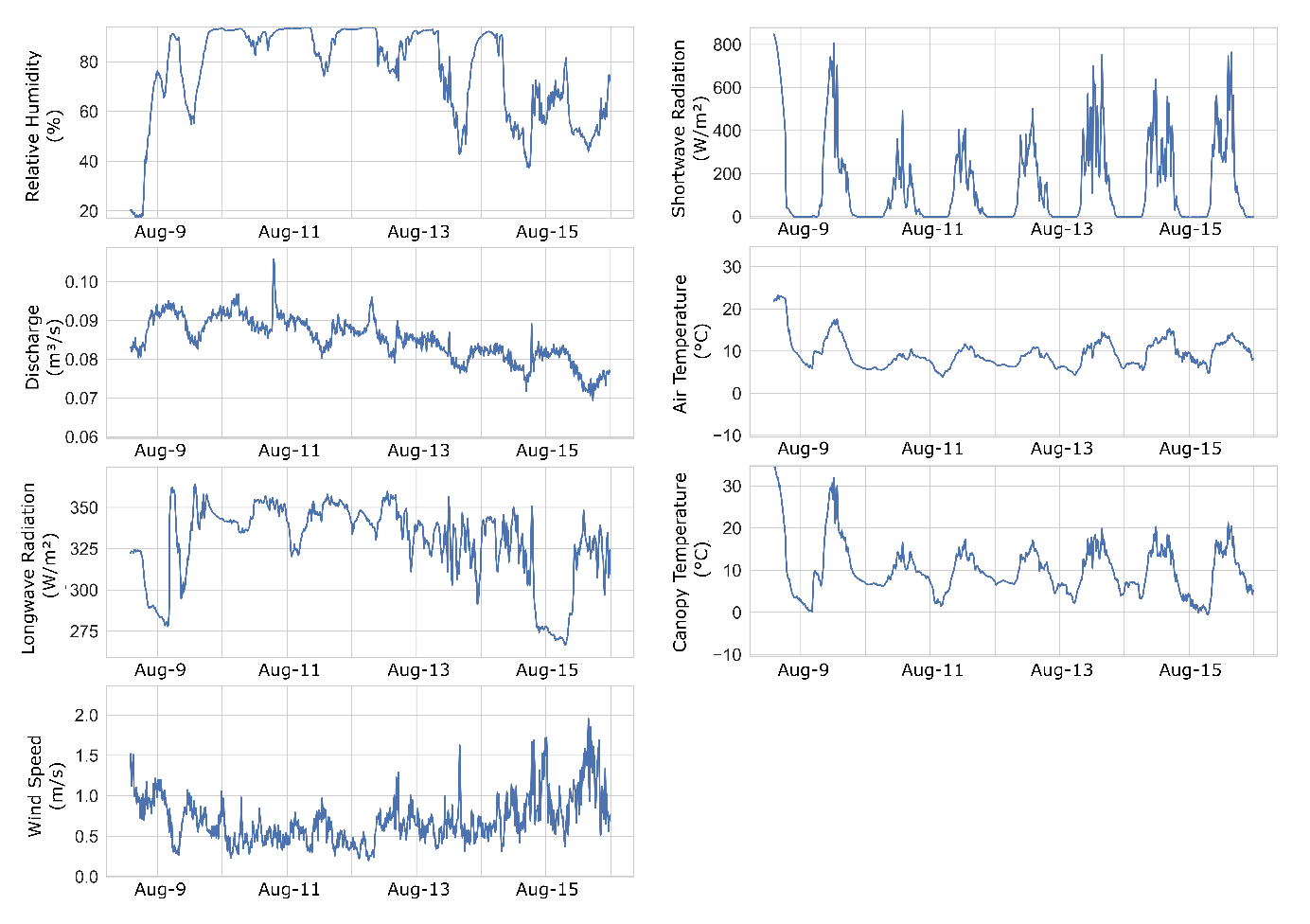


**S4. Stage-discharge rating curve for GS2**


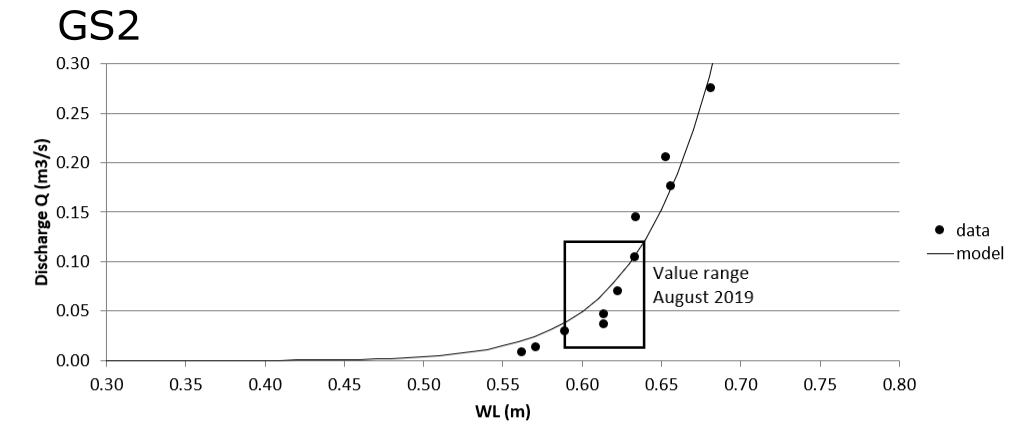

Supplement: Supplementary file 1 — Data S1. Choosing a ML‐model for residual prediction. Data S2. PCA SHAP values, HFLUX predictions and ML‐model prediction plots. Data S3. Timeseries of variables for 8–16 August 2019. Data S4. Stage‐discharge rating curve for GS2. [file HYP-36-0-s001.docx]
